# METTL18 functions as a Phenotypic Regulator in Src-Dependent Oncogenic Responses of HER2-Negative Breast Cancer

**DOI:** 10.7150/ijbs.96487

**Published:** 2024-09-03

**Authors:** Han Gyung Kim, Ji Hye Kim, Kyung-Hee Kim, Byong Chul Yoo, Sung-Ung Kang, Young Bong Kim, Sangmin Kim, Hyun-June Paik, Jeong Eon Lee, Seok Jin Nam, Narayanan Parameswaran, Jeung-Whan Han, Balachandran Manavalan, Jae Youl Cho

**Affiliations:** 1 Department of Integrative Biotechnology, Sungkyunkwan University, Suwon 16419, Republic of Korea.; 2 Research Institute, National Cancer Center, Goyang 10408, Republic of Korea.; 3 Neuroregeneration and Stem Cell Programs, Institute for Cell Engineering, Johns Hopkins University School of Medicine, Baltimore, MD 21205, USA.; 4 Department of Bio-industrial Technologies, Konkuk University, Seoul 05029, Republic of Korea.; 5 Breast Cancer Center, Samsung Medical Center, Seoul 06351, Republic of Korea.; 6 Department of Surgery, Pusan National University Yangsan Hospital, Pusan National University School of Medicine, Yangsan 50612, Republic of Korea.; 7 Division of Breast, Department of Surgery, Samsung Medical Center, Sungkyunkwan University School of Medicine, Seoul 06351, Republic of Korea.; 8 Department of Physiology and Division of Pathology, Michigan State University, East Lansing, MI 48824, USA.; 9 Research Center for Epigenome Regulation, School of Pharmacy, Sungkyunkwan University, Suwon 16419, Republic of Korea.

**Keywords:** METTL18, HSP90, actin, Src kinase, RPL3 methylation, histidine methyltransferase, metastasis, migration, invasion, breast cancer

## Abstract

Methyltransferase-like (METTL)18 has histidine methyltransferase activity on the RPL3 protein and is involved in ribosome biosynthesis and translation elongations. Several studies have reported that actin polymerization serves as a Src regulator, and HSP90 is involved in forming polymerized actin bundles. To understand the role of METTL18 in breast cancer and to demonstrate the importance of METTL18 in HER-2 negative breast cancer metastasis, we used biochemical, molecular biological, and immunological approaches *in vitro* (breast tumor cell lines), *in vivo* (tumor xenograft model), and in samples of human breast tumors. A gene expression comparison of 31 METTL series genes and 22 methyltransferases in breast cancer patients revealed that METTL18 is highly amplified in human HER2-negative breast cancer. In addition, elevated levels of METTL18 expression in patients with HER2-negative breast cancer are associated with poor prognosis. Loss of METTL18 significantly reduced the metastatic responses of breast tumor cells *in vitro* and *in vivo*. Mechanistically, METTL18 indirectly regulates the phosphorylation of the proto-oncogene tyrosine-protein kinase Src and its downstream molecules in MDA-MB-231 cells via METTL18-mediated RPL3 methylation, which is also involved in determining HSP90 integrity and protein levels. In confocal microscopy and F/G-actin assays, METTL18 was found to induce actin polymerization via HSP90. Molecular events involving METTL18, RPL3, HSP90, and actin polymerization yielded Src phosphorylated at both tyrosine 419 and tyrosine 530 with kinase activity and oncogenic functions. Therefore, it is suggested that the METTL18-HSP90-Actin-Src regulatory axis plays critical oncogenic roles in the metastatic responses of HER2-negative breast cancer and could be a promising therapeutic target.

## Introduction

Breast cancer is a common cause of cancer-related death in women 1. It is a heterogeneous disease that can be distinguished into four subtypes based on the biomarkers estrogen receptor (ER), progesterone receptor (PR), and human epidermal receptor 2 (HER2) biomarkers. These subtypes include luminal A (HER2^-^), luminal B (ER^+^), HER2 (HER2^+^), and triple-negative breast cancer (TNBC; PR^-^, ER^-^, and HER2^ -^) 2-4. The most common breast cancer subtype is luminal A, accounting for 54.3% of new patients of breast cancer 5. This subtype has a high potential for bone metastasis.^6^ TNBC, which accounts for 10-15% of breast cancers, is another subtype without HER2 and is aggressive, with the higher recurrence rate than all HER2^+^ breast cancers 7,8. Additionally, metastatic breast cancer, which develops in about 30% of women with early-stage breast cancer, contributes to up to 80% of breast cancer-related deaths, whereas localized carcinoma accounts for only 10% 9. Therefore, it is necessary to use present knowledge about both tumorigenesis and metastatic events in breast cancer to identify potential therapeutic targets. It is also important to understand that different subtypes of breast cancer progress through distinguished mechanisms, and these differences must be considered when diagnosing and treating cancers.

METTL18 is a member of the methyltransferase-like protein (METTL) family. It is also known as C1orf156 or arsenic-transactivated protein 2, AsTP2, and is a human homolog of yeast histidine methyltransferase, YIL110W 10. While histidine methylation is a rare protein modification found only in a few proteins such as β-actin 11, S100A9 12, myosin 13, myosin light chain kinase 14, and ribosomal protein RPL3 10, a recent study has shown that protein methylation on histidine residues is common in intracellular proteins in mammalian cells 15. However, research in this area is limited by a lack of effective reagents for the probing protein-histidine methylation 16. SET domain containing 3 (SETD3) has recently been recognized as an actin histidine methyltransferase. It plays a role in controlling replication and pathogenesis in various mouse models of enterovirus infection and is also involved in the regulation of smooth muscle contractility associated with primary dystocia 17-19. Moreover, it has been reported that METTL18 monomethylates the histidine (His)-245 residue of RPL3 and plays an essential function in ribosome biosynthesis and mRNA translation 20. However, the role of METTL18 in breast cancer remains unknown and requires further study.

Tyrosine kinases play an essential role in regulating signal transduction processes, morphology, cell growth, migration rate, metastasis, and programmed cell death in many tumor cells, and their expression is highly elevated 21. Src, a tyrosine kinase, is involved in multiple tumorigenic responses and is present in approximately half of all cancerous tumors in the brain, lung, colon, liver, stomach, and breast 22,23. In the absence of stimulation, the Src kinase exists in a closed (or autoinhibited) state due to an intramolecular interaction between the phosphorylated Y530 residue and the SH2 domain 24. Generally, dephosphorylation of p-Y530 by phosphatases (such as receptor protein tyrosine phosphatase α) or downregulation of CSK (which is responsible for the phosphorylation of Y530) switches Src to an open, activated state 25. Thus p-Src (Y419) and p-Src (Y530) are the active and inactive forms, respectively, in the canonical Src regulatory pathway 26. Our previous studies showed that the inhibition of methylation by adenosine dialdehyde (AdOx) 27,28 reduced the Src kinase activity and regulated metastatic responses in breast cancer 29. As AdOx is a methylation inhibitor, we expected a methyltransferase to be involved in Src regulation and the metastatic ability of breast cancer. Interestingly, the expression of METTL18 was increased in HER2-negative breast cancers, and this increase was associated with the upregulation of p-Src. Therefore, in this study, we examined the role of METTL18 as a crucial component of metastatic responses by breast tumor cells and carefully dissected the underlying mechanisms involved in Src regulation by METTL18.

## Materials and methods

### Materials and antibodies

Anti-METTL18 (Atlas HPA035314), anti-phospho-tyrosine (Upstate 05-321), anti-PIMT (ab 70559), and anti-RPL3 (Proteintech 66130) antibodies were used. Anti-Src (# 2109), anti-phospho-Src-Y416 (# 2101), anti-phospho-Src-Y527 (# 2105), anti-Syk (# 2712), anti-phospho-Syk (# 2711), anti-JAK2 (# 3230), anti-phospho-JAK2 (# 3771), anti-ATF2 (# 9226), anti-phospho-ATF2 (# 24329), anti-p85 (# 4292), anti-phospho-p85 (# 4228), anti-STAT3 (# 4904), anti-phospho-STAT3 (# 9145), anti-c-Jun (# 9165), anti-phospho-c-Jun (# 9164), anti-c-Fos (# 2250), anti-phospho-c-Fos (# 5348), anti-p65 (# 8242), anti-phospho-p65 (# 3039), anti-p50 (# 12540), anti-phospho-p50 (# 4806), anti-HER2 (# 2242), anti-Myc (# 2276), anti-PRMT1 (# 2449), anti-Flag (# 8146), anti-HSP90 (# 8165), anti-tubulin (# 2128), HRP-conjugated anti-mouse IgG (#91196), and anti-β-actin (# 4967) were purchased from Cell Signaling (Danvers, MA, USA). Anti-GAPDH (sc 166545), anti-phospho-c-Src-419 (sc 81521), anti-phospho-Src-530 (sc 166860), and anti-HA (sc 7392) were obtained from Santa Cruz Biotechnology. Anti-pan-methyl-histidine antibody (A17948) was from Abclonal (Woburn, MA, USA). siRNAs to human EEF2KMT, PIMT, PRMT1, GRWD1, RPL3, METTL18, HSP90AA1, actin, and scramble were purchased from Genolution (Seoul, Korea), and the sequences are shown in Table S1. Phalloidin, VER-15508, S-adenosyl-L-methionine, and cytochalasin B were purchased from Sigma Chemical Co. (St. Louis, MO, USA).

### Construction of expression vectors and virus production

The wild-type construct of Myc-tagged METTL18 was obtained by a culture method using competent *Escherichia coli* (DH5α). The pcDNA-HA and pcDNA-HA-tagged c-Src constructs (HA-Src-WT) were prepared as reported previously 30. The mutated constructs were produced using a QuikChange site-directed mutagenesis kit. All construct sequences were confirmed using the ABI 3730xl System from Macrogen, Inc. (Seoul, Korea). Lentiviruses containing shScramble, shMETTL18-2974, shMETTL18-2975, shSrc-#1, or shSrc-#2 were expressed by transfection into HEK293T cells with polyethylenimine (Sigma), pMD2.G (Addgene 12259), and psPAX2 (Addgene 12260), which were a gift from K. Yoon. The shRNA targeting regions of METTL18 (TRCN0000232975-shMETTL18) and Src (TRCN0000038149-shSrc-#1 and TRCN0000038150-shSrc-#2) were obtained from Sigma. Primer sequences for shMETTL18, shSrc-#1, and shSrc-#2 are listed in Table S2. The viral supernatants were collected after 48 h.

### Cell culture

The breast cancer cell line MDA-MB-231 was purchased from ATCC (Manassas, VA, USA) and was maintained in DMEM (SH30249, Hyclone, USA) supplemented with 10% fetal bovine serum (SH30919, Hyclone, USA), along with penicillin and streptomycin, at 37°C in a humidified atmosphere with 5% CO_2_. Knock down cells were infected by lentiviral particles that expressed shSrc or shMETTL18 with polybrene (8 μg/ml). Cells were selected by puromycin (1 μg/ml) for 48 hours after infection. Knock-down levels of target protein were analyzed by Western blotting.

### METTL18 expression in breast cancer subtypes

The mRNA expression levels of METTL in breast cancers with HER2 expression were analyzed using the breast invasive carcinoma dataset from cBioPortal (TCGA, PanCancer Atlas). The gene expression of METTL18 in breast cancer with and without HER2 expression was compared with that in normal breast tissue using RNA-sequencing (RNA-seq) data from GEPIA2 (http://gepia2.cancer-pku.cn/ #index) 31 from HER2-negative tumors (n=550), HER2-negative normal tissue (n=291), HER2-positive tumors (n=260), and HER2-positive normal (n=291) tissue samples from the Cancer Genome Atlas (TCGA) and the Genotype-Tissue Expression project. Transcriptional expression levels of METTL18 were normalized and measured using TPM (transcripts per million), and the y-axis log-scale was presented as log2(TPM+1). TCGA data were used to compare the gene expression of METTL18 and other methyltransferases in HER2-positive and -negative breast cancers. Differences in the expression levels of METTL18 in metastatic and non-metastatic patients were analyzed using a dataset from the Metastatic Breast Cancer Project (Provisional cBioportal, Metastatic Breast Cancer Project, https://identifiers.org/cbioportal:brca_mbcproject_wagle_2017 (2020)]). The analysis was conducted using the RSEM, an accurate transcript quantification software tool for RNA-sequencing data as reported previously 32.

### Kaplan-Meier analysis

The Kaplan-Meier Plotter (http://www.kmplot.com) is an online public database for assessing the association between gene expression levels and survival outcomes in breast cancer 33. It was used to analyze overall survival and distant metastasis-free survival (DMFS).

### Preparation of breast tumor tissues

Human breast tumor tissue and information on the overall survival of patients were obtained from Samsung Medical Center (Seoul, Korea). We used immunoblotting to assess METTL18, p-Src(Y419), Src, and β-actin levels in whole lysates from these tumor tissues. Approval for this study was granted by the Institutional Review Board at Sungkyunkwan University Medical School.

### Protein isolation and immunoblotting

For lysis, cultured cells (5 × 10^6^ MDA-MB-231 cells/ml) or tissues (10 mg each of normal or tumor tissues) were lysed with LIPA buffer with protease inhibitor cocktail. The protein levels in whole lysates were analyzed by Western blotting. For immunoblotting, proteins were resolved on either 10% or 12% SDS-polyacrylamide gels (SDS-PAGE) and then transferred to polyvinylidene difluoride membranes that were blocked in Tris-buffered saline with 3% bovine serum albumin. Afterward, the membrane was incubated with a primary antibody corresponding to the interesting protein. The tyrosine residues 416 and 527 (Y416 and Y527) of chicken Src are conserved across various species and correspond to Y419 and Y530 in humans, respectively. Therefore, for the detection of p-Src Y419 and p-Src Y530 in human cell lines and breast cancer patient tissues, we utilized anti-p-Src Y416 (Cell Signaling, #2101) and anti-p-Src Y527 (Cell Signaling, #2105), respectively. These antibodies exhibit cross-reactivity across species, including human, mouse, rat, and chicken. Following the primary antibody treatment, the membrane underwent three washes with TBST before being incubated with a secondary antibody. Subsequently, the proteins were detected using EzWestLumi plus (ATTO, Tokyo, Japan).

### Immunoprecipitation assay

Immunoprecipitation was performed with equal amounts of protein. We added 5 μl of anti-Myc, anti-HA, anti-actin, or anti-p-Src Y527 (Cell signaling, #2105) and incubated this mixture overnight at 4°C. The anti-p-Src Y527 was employed to immunoprecipitate the human p-Src Y530. In addition, protein G or A Sepharose 4 fast flow was added to the mixture, which was incubated for 4 hours at 4°C to bind the antibody-protein complex and beads. Cell lysates and immunoprecipitants were analyzed through immunoblotting. To prevent IgG-heavy chain blotting, Mouse TrueBlot® ULTRA: anti-mouse IgG-HRP and Rabbit TrueBlot®: anti-rabbit IgG HRP (Rockland Immunochemicals, Pottstown, PA, USA) were used as secondary antibodies.

### Determination of metastatic potential *in vitro*

In wound healing (cell migration) assays, wounds were created in monolayered MDA-MB-231 cells by scratching them with a p200 tip. In the invasion assay, cells were cultured on a Matrigel layer with a gradient of FBS. Invasive cells were fixed with 4% formaldehyde, and the cells were counted after staining with hematoxylin and eosin.

### Gelatin zymography

A sample containing 10 mg of protein was obtained from cell lysates and mixed with a non-reducing sample buffer. The mixture was loaded onto a polyacrylamide gel incorporating gelatin (0.25 mg/ml; Sigma) for 2 hours at 100 V. Then, the gel was incubated in renaturing buffer for 36 hours at 37°C. To detect the activation of gelatinases (MMP2 and MMP9), each gel was stained with Coomassie brilliant blue and destained using methanol/acetic acid (30%/10% v/v) solutions.

### Tumor xenografts and PET/CT

Tumor xenograft models were prepared by injecting subcutaneously 5×10^6^ of the indicated cells into the backs of six-week-old *nu/nu* mice (Harlan Sprague-Dawley, IN, USA). Body weights and tumor volumes were measured every day. To determine metastasis, PET/CT scanning was performed with MDA-MB-231 cell-injected mice. MMWKS Vista-CT software from the scanner manufacturer was used for presenting PET and CT images. For calculation of the tumor volume (mm^3^), all tumor tissues were subsequently estimated the volume by utilizing the formula 34: *V = 0.5 × D × d^2^*. The long (*D*) and short axes (*d*) of the tumors were determined using calipers. All animal experimental processes were approved by the Institutional Animal Care and Committee of the National Cancer Center (Ilsan, Korea).

### QuantSeq 3' mRNA-Seq

Total RNA was isolated using TRIZOL (GIBCO-BRL, Grand Island, NY, USA). E-Biogen Inc. (Seoul, Korea) performed the RNA sequencing and data analysis. The quality of RNA was validated using an Agilent 2100 bioanalyzer (Agilent Technologies, Amstelveen, Netherlands). Library construction was conducted according to the manufacturer's protocol utilizing a QuantSeq 3' mRNA-Seq library prep kit (Lexogen, Inc., Greenland, NH, USA). High-throughput sequencing was carried out using single-end 75 sequencings reads on a NextSeq 500 (Illumina, Inc., San Diego, CA, USA). Gene ontology term enrichment was performed using DAVID for selected genes.

### Protein phosphorylation profiling

Total proteins were prepared without trypsin. Full Moon Biosystems, Inc. (Sunnyvale, CA) designed the Phospho Explorer Antibody Array (PEX100), which contained 1318 antibodies. E-Biogen Inc. (Seoul, Korea) performed the antibody array experiment and data analysis following the manufacturer's protocol.

### Confocal microscopy analysis

MDA-MB-231, HAP-1 WT, and HAP-1 METTL18 knockout cells transfected with METTL18, shMETTL18, siMETTL18, siHSP90, or siActin were plated in 12-well plates over sterile coverslips at a density of 2 × 10^5^ cells. The cells were rinsed twice with 1 ml of phosphate-buffered saline (PBS) and fixed with 3.7% paraformaldehyde. Following three washes with PBS, the coverslips were blocked using 1% bovine serum albumin (BSA). Dapi (1:1000), rhodamine phalloidin (Invitrogen, 1:40), or the indicated antibodies were administered to the cells for staining of the nucleus, polymerized actin, or specific target proteins, respectively. As an exception, since the anti p-Src Y416 (Cell Signaling, #2101) has cross-reactivity with the human counterpart, it was employed to detect human p-Src Y419. The stained cells were detected with confocal microscopy (LSM800, Zeiss).

### Liquid chromatography-mass spectrometry/mass spectrometry (LC-MS/MS) analysis

Immunoprecipitation was performed with anti-Myc in Myc-METTL18-overexpressed MDA-MB-231 cells. Immunoprecipitates were resolved by SDS-PAGE for protein size separation, and the gels were subsequently stained with an EzWay silver staining kit (Koma Biotech, Seoul, Korea). Bands of interest were cut from the gels and analyzed using a Q ExactiveTM hybrid quadrupole-orbitrap mass spectrometer (Thermo Fisher Scientific) coupled with an Ultimate 3000 RSLCnano system (Thermo Fisher Scientific) 35.

### Proximity ligation assay (PLA)

PLAs were performed using NaveniFlex MR assay reagents according to the manufacturer's protocols (Navinci Diagnostics AB). MDA-MB-231 cells were transfected by METTL18 blocked with a blocking buffer for 1 hour at 37℃. Next, primary antibodies against phospho-Src Y416 (rabbit polyclonal antibody, Cell Signaling, #2101) and phospho-Src Y530 (mouse monoclonal antibody, Santa Cruz, sc-166860) were incubated with the cells for 1 hour at 37℃. The anti-p-Src Y416 was utilized to detect human p-Src Y419. After primary antibody incubation, the cells were washed with 1x TBS-T three times. Next, probes were added to the cells and incubated for 1 hour at 37℃. Then, cells were washed with 1x TBS-T three times, and enzyme A (PLA probe conjugation) was added for 1 hour at 37℃, followed by incubation with enzyme B (probe connecting) for 30 min at 37℃. Finally, the cells were treated with buffer C Atto 488 and enzyme C (amplification) for 90 min at 37℃. After PLA, the nuclei were stained with DAPI (Sigma) for 5 min at room temperature and were detected with a Zeiss LSM700 confocal microscope. Specific individual protein-protein interactions can be seen as green dots. For all experimental conditions, at least three images were acquired.

### *In vitro* methylation assay

METTL18 methyltransferase activity was measured using an MTase-Glo^TM^ Methyltransferase assay (Promega, Madison, WI) following the manufacturer's protocol. Immunoprecipitated METTL18 served as an enzyme source, while immunoprecipitated RPL3 was used as a substrate.

### *In vitro* Src kinase assay

Src kinase activity was measured using an ADT-Glo^TM^ kinase assay (Promega, Madison, WI) and Src kinase enzyme system (Promega, Madison, WI) following the manufacturer's protocol. Immunoprecipitated Src was used as an enzyme source, and immunoprecipitated Myc-PI3K or peptide (KVEKIGEGTYGVVYK) designed from p34cdc2 was used as a substrate.

### Comparisons of protein and mRNA expression levels

We used the breast invasive carcinoma (TCGA PanCancer Atlas) dataset for comparisons of protein levels of the METTL series and HSP90AA1. TCGA-RPPA-protein expression Z-scores (mass spectrometry by CPTAC) relative to normal samples (log RNA seq V2 RSEM) were obtained from c-BioPortal (https://www.cbioportal.org/). Correlation of the protein levels of METTL series and the protein levels of HSP90AA1 was analyzed by calculating the Pearson's correlation.

### Statistics and reproducibility

All data are shown as the mean ± SD from a minimum of three independent experiments. Statistical analysis entailed conducting one-way analysis of variance (ANOVA), subsequently employing Tukey's post-hoc test for comparison. All P values ≤ 0.05 were regarded as statistically significant. GraphPad Prism version 8.0 (GraphPad Software) was used for the data analyses.

## Results

### Clinical relevance of METTL18 in human HER2-negative breast cancer

**Figure 1:**
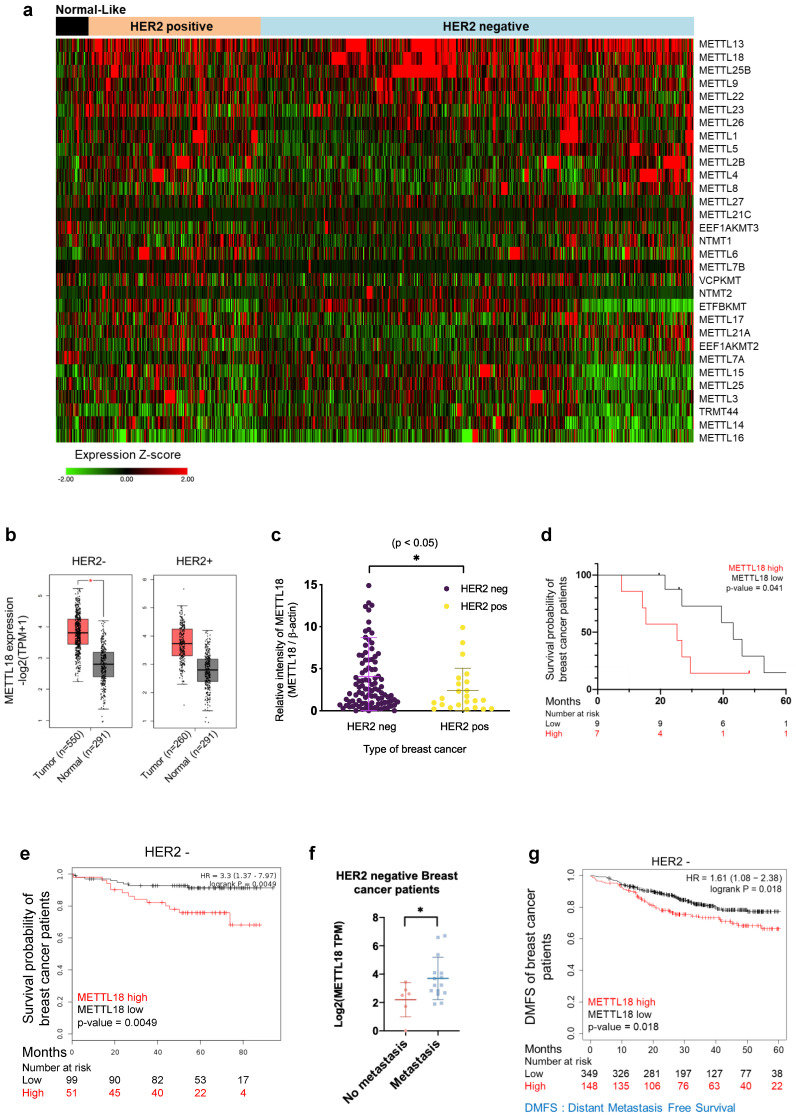
**Clinical relevance of METTL18 in human HER2-negative breast cancer.** (A) Heatmap of expression profiles of METTL18 and 30 METTL series genes. Gene expression was analyzed in normal-like, HER2-positive, and HER2-negative breast cancer samples in the TCGA database (n=1080). (B) Gene expression profile of METTL18 in breast cancer tissues and normal tissues. We used the GEPIA2 dataset and classified samples into four groups: normal tissues from HER2-negative patients (n=291), tumor tissues from HER2-negative patients (n=550), normal tissue from HER2-positive patients (n=291), and tumor tissues from HER2-positive patients (n=260). (C) Protein expression profile of METTL18 in breast tumor tissue from Samsung's cohort (n=150). Patients were divided into HER2-positive and HER2-negative. (D) Overall survival time (months) of Samsung's cohort breast cancer patients with low or high levels of METTL18 (median cutoff). (E) Kaplan-Meier curve showing the survival probability of HER2-negative breast cancer patients with high or low expression of METTL18 (median cutoff). (F) Gene expression of METTL18 in HER2-negative breast cancer patients with or without metastasis. Database from the Metastatic Breast Cancer Project [Provisional, Feb 2020] was used. (G) DMFS of HER2-negative breast cancer patients with low or high expression of METTL18 (median cutoff) (GSE25066). * *P* < 0.05, ** *P* < 0.01, ns: not significant.

To elucidate the biological role of METTL18, we compared the mRNA expression level of METTL18 with 30 other METTL series genes and 22 methyltransferases using TCGA data, which are classified by breast cancer subtype. The METTL18 gene showed a breast cancer subtype-specific expression distribution, with high amplification in HER2-negative breast cancer patients (Fig. 1A and Fig. S1A and B). METTL18 was the third most highly expressed among the 31 METTL genes and 23 methyltransferases examined (Fig. 1A and Fig. S1A). METTL13 and SETDB1 were the two more highly expressed genes (Fig. 1A and Fig. S1A). However, METTL13 expression did not significantly differ between HER2-negative and HER2-positive breast cancers (Fig. S1B), and SETDB1 is a well-known potential modulator of breast cancer metastasis 36. We analyzed METTL18 gene levels in normal and tumor tissues from breast cancer patients using the publicly available gene expression profiling interactive analysis 2 (GEPIA2) dataset 31. In the HER2-negative subtypes, METTL18 was more highly expressed in tumors than in normal tissues (Fig. 1B). But HER2-positive patients showed no significant difference in METTL18 expression between normal and tumor tissues (Fig. 1B). To demonstrate the METTL18 pattern in Korean breast cancer patients, tissue of 150 Korean breast cancer patients (*Samsung's cohort*) was used to evaluate its expression. Again, METTL18 protein expression was higher in tumor tissue from HER2-negative patients but not in HER2-positive tumors (Fig. 1C). The high expression of METTL18 in HER2-negative patients was associated significantly with shorter overall survival (Fig. 1D). Similarly, Kaplan-Meier plots indicate that HER2-negative patients with elevated METTL18 levels had a reduced survival probability compared to those with lower METTL18 levels (p=0.0049) (Fig. 1E). The association between METTL18 and metastasis was further investigated using the database of patient samples from the Metastatic Breast Cancer Project (Provisional cBioportal, Metastatic Breast Cancer Project). METTL18 was abundantly expressed in patients with HER2-negative breast cancer with metastases (Fig. 1F). Consistently, a significantly lower level in DMFS was observed in HER2-negative patients with high METTL18 levels (p=0.012) (Fig. 1G). Conversely, a HER2-positive patient with high METTL18 expression had significantly higher than average overall survival (p=0.02) and DMFS (p=0.03) (Fig. S1C and D). These results suggest that METTL18 is positively involved in the metastasis of HER2-negative breast cancer.

### Effects of METTL18 on breast cancer metastasis

**Figure 2:**
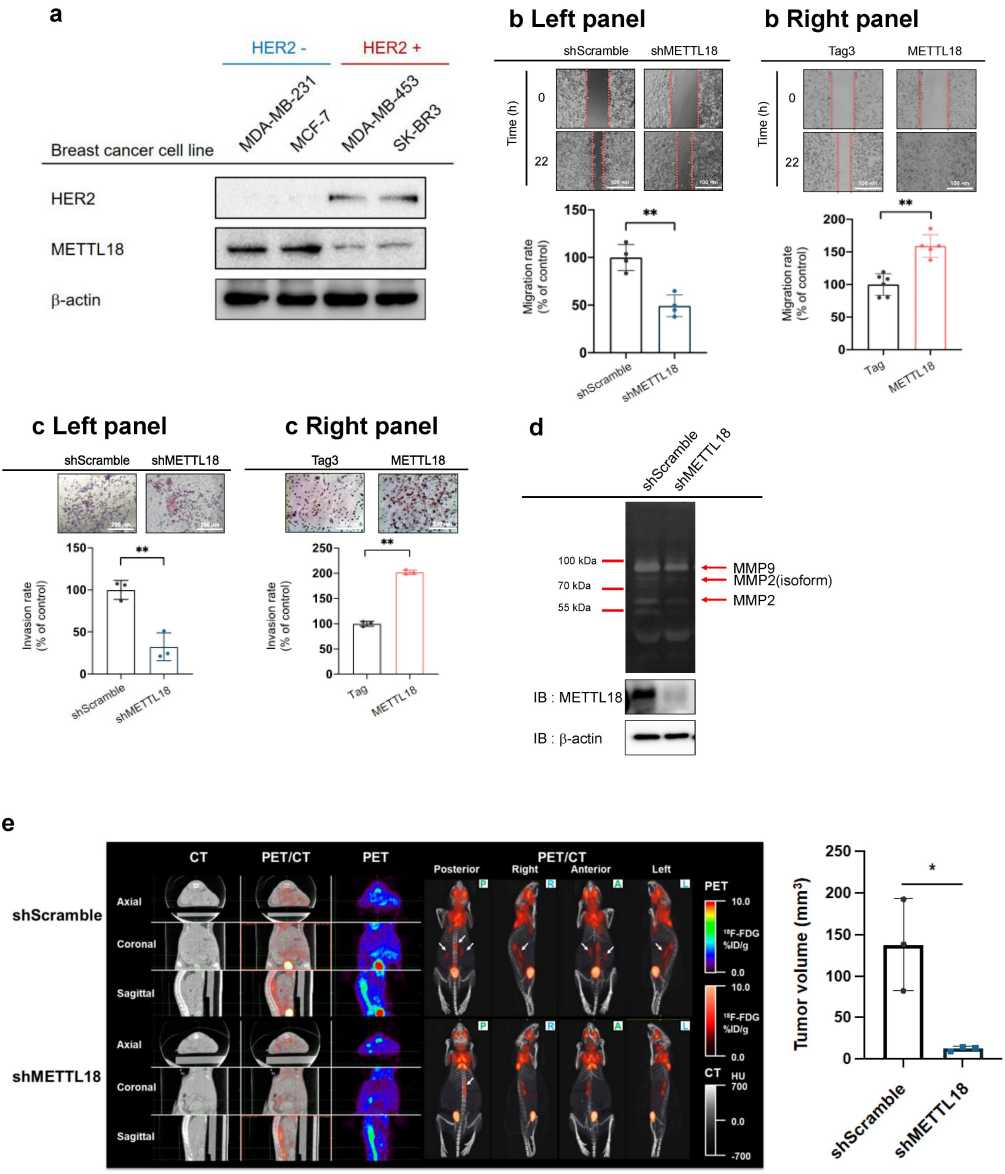
** Effect of METTL18 on metastatic responses in breast cancer cells *in vitro* and* in vivo.*** (A) Protein expression of METTL18 in breast cancer cell lines. Protein levels of HER2, METTL18, and β-actin were identified by Western blotting. (B) Migration capacity of METTL18-knockdown (left panel) and METTL18-overexpressing (right panel) MDA-MB-231 cells. Knockdown and overexpression of METTL18 were confirmed by western blotting (Fig. S2A). The Tag3 vector, utilized for overexpressing METTL18, served as the vehicle control. ImageJ measured the migration range. (C) Invasive capacity of METTL18 knockdown (left panel) and METTL18-overexpressing (right panel) MDA-MB-231 cells. Knockdown and overexpression of METTL18 were confirmed by western blotting (Fig. S2A). The Tag3 was used as a control of METTL18 overexpression. (D) Enzyme activities of MMP-2 and MMP-9 in METTL18-knockdown MDA-MB-231 cells. (E) Metastasis of tumors in xenograft mice intravenously injected with shScramble- or shMETTL18-transfected MDA-MB-231 cells. The white arrows indicate metastatic tumors, visualized via ^18^F-FDG-PET/CT scans. The blot shown in (A) and (D) is a representative image of three independent Western blot experiments. * *P* < 0.05; ** *P* < 0.01.

To identify the expression level of METTL18 in various subtypes of breast cancer cell lines, the protein levels of METTL18 were examined in MDA-MB-231 (TNBC cell line), MCF-7 (luminal A type cell line), and HER2-enriched cell lines including MDA-MB-453 and SK-BR3. The protein levels of METTL18 were elevated in HER2-negative cells than in HER2-positive cell lines (Fig. 2A). The overexpression of METTL18 in MDA-MB-231 cells increased the migration and invasion rates, and knockdown of METTL18 reduced the migration and invasion capabilities of MDA-MB-231 cells (Fig. 2B, C). In addition, Fig. 2D shows that the knockdown of METTL18 suppressed the activity of MMP-9 and MMP-2, which are enzymes responsible for degrading the extracellular matrix (ECM) 37. Since ECM remodeling promotes the migration and invasion of tumor cells, the activity of MMP-9 and MMP-2 was employed as molecular biomarkers supporting the invasion and migration assay results. Moreover, the inhibitory effect on migration was also observed in MCF-7 cells expressing shMETTL18 (Fig. 2SB). Taken together, these results support the critical role of METTL18 in the metastatic response of HER2-negative breast cancer cells. Following that, we examined the role of METTL18 in metastasis in a murine model. Metastasis was detected using ^18^F-FDG-PET/CT scans, and the results were consistent with our *in vitro* findings of decreased by knockdown of METTL18 without a change in body weight (Fig. 2E Left panel, Fig. S3). Statistical analysis with quantitative measurements also supported this pattern (Fig. 2E Right panel).

### Effect of METTL18 on the activation of the Src kinase and its downstream pathway

**Figure 3:**
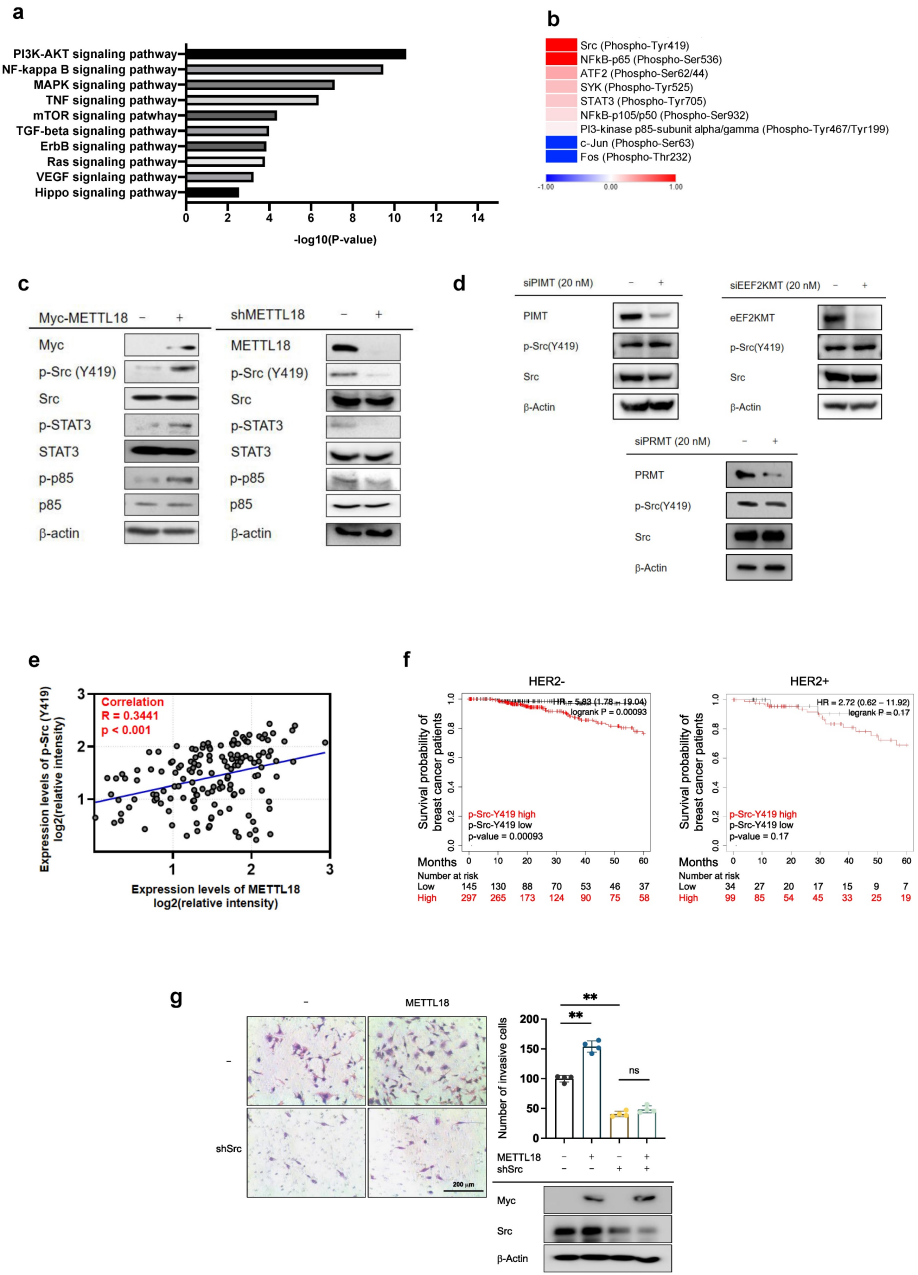
** Effect of METTL18 on the Src kinase signaling pathway.** (A) Significantly altered pathways in the RNA-seq analysis of shMETTL18 #2795-expressing MDA-MB-231 cells (cutoff for p-value < 0.01). (B) Heatmap of phospho-protein array data. Samples were prepared from METTL18-overexpressing MDA-MB-231 cells. (C) Immunoblotting for the phospho- and total forms of tyrosine kinases (Src, STAT3, and p85) in Myc-METTL18-overexpressing or shMETTL18-expressing MDA-MB-231 cells. Transfection efficacy of the Myc-METTL18 construct and shMETTL18 was verified by immunoblotting with anti-Myc and anti-METTL18, respectively. β-actin was used as the loading control. (D) Western blotting results for the phospho- and total protein levels of Src in siPIMT (20 nM)-, siEEF2KMT (20 nM)-, and siPRMT1 (20 nM)-transfected MDA-MB-231 cells after 48 hours. (E) Scatterplot showing the correlation between p-Src and METTL18 in breast cancer patients from Samsung's cohort. Source data for the scatterplot were obtained from immunoblotting (Fig. S5). *P*-values and the correlation coefficient (*R*) were calculated using GraphPad Prism. (F) Kaplan-Meier curve showing the survival probability of HER2-negative breast cancer patients with high or low expression of p-Src Y419 (best cutoff). (G) Invasive capacity of MDA-MB-231 cells transfected with Myc, Myc-METTL18, shScramble, or shSrc. The transfection efficacy of the plasmids and shRNA was verified by Western blotting with anti-Myc and anti-Src. The blots shown in (C), (D), and (G) are representative images of three independent Western blot experiments. ns: not significant; * *P* < 0.05; ** *P* < 0.01; ## *P* < 0.01.

To identify the molecular mechanisms of METTL18-dependent metastatic responses, RNA-seq analysis was performed with shMETTL18-expressing MDA-MB-231 cells. The results indicated the pivotal role of METTL18 in PI3K-AKT and NF-κB signals (Fig. 3A). In the phospho-protein array, the phosphorylation of Src (p-Tyr418) and p65 (p-Ser536) was markedly increased by METTL18 overexpression (Fig. 3B). Src kinase is a well-known upstream regulator of the PI3K-AKT and NF-κB pathways 38,39. Therefore, we hypothesized that one of the main targets of METTL18 is Src. METTL18 overexpression increased the phosphorylation levels of the Src kinase at tyrosine 419, and METTL18 deficiency decreased it, which supports our hypothesis (Fig. 3C). Moreover, METTL18 altered the p-levels of other molecules associated with the PI3K-AKT and NF-κB pathways, such as STAT3, p85, p65, and p50 (Fig. 3C and Fig. S4A). However, METTL18 overexpression did not upregulate p-c-Jun, p-c-Fos, p-Syk, p-JAK2, or p-ATF2 (Fig. S4A). Interestingly, METTL18 knockdown affected p-Src levels only in MBA-MB-231 cells and not in SK-BR3 or MDA-MB-453 cells, indicating that Src regulation by METTL18 is limited to HER2-negative breast cancer (Fig. S4B). Finally, the isoaspartyl, lysine, and arginine methyltransferases (PIMT, EEF2KMT, and PRMT1, respectively) did not decrease p-Src levels (Fig. 3D), implying that METTL18 has specificity to Src regulation. In breast cancer tissue from the Samsung cohort, phosphorylated-Src levels consistently correlated with METTL18 expression (Fig. 3E and Fig. S5). However, the mRNA expression levels of Src remained unchanged in breast cancer (Fig. S6), suggesting that METTL18 regulates Src at the post-translational level. To confirm the role of Src in METTL18-mediated metastasis regulation, we investigated the effect of Src on tumorigenesis and compared it with that of METTL18.

Like METTL18, survival probability was diminished in HER2-negative breast cancer patients exhibiting heightened phosphorylation of Src at Y419. In contrast, survival probability in patients with HER2-positive breast cancer showed the opposite pattern (Fig. 3F). Like shMETTL18, tumorigenic responses such as proliferation, invasion, migration, and clonogenic capacity were inhibited by Src knockdown in MDA-MB-231 cells (Fig. S7A, B, and C). Crucially, METTL18 overexpression significantly increased the invasive ability of shScramble-expressing MDA-MB-231 cells but not shSrc-expressing cells, suggesting that Src is a significant factor involved in the regulation of METTL18-mediated tumorigenesis (Fig. 3G).

### Contribution of RPL3 to the METTL18-Src regulatory mechanism

**Figure 4:**
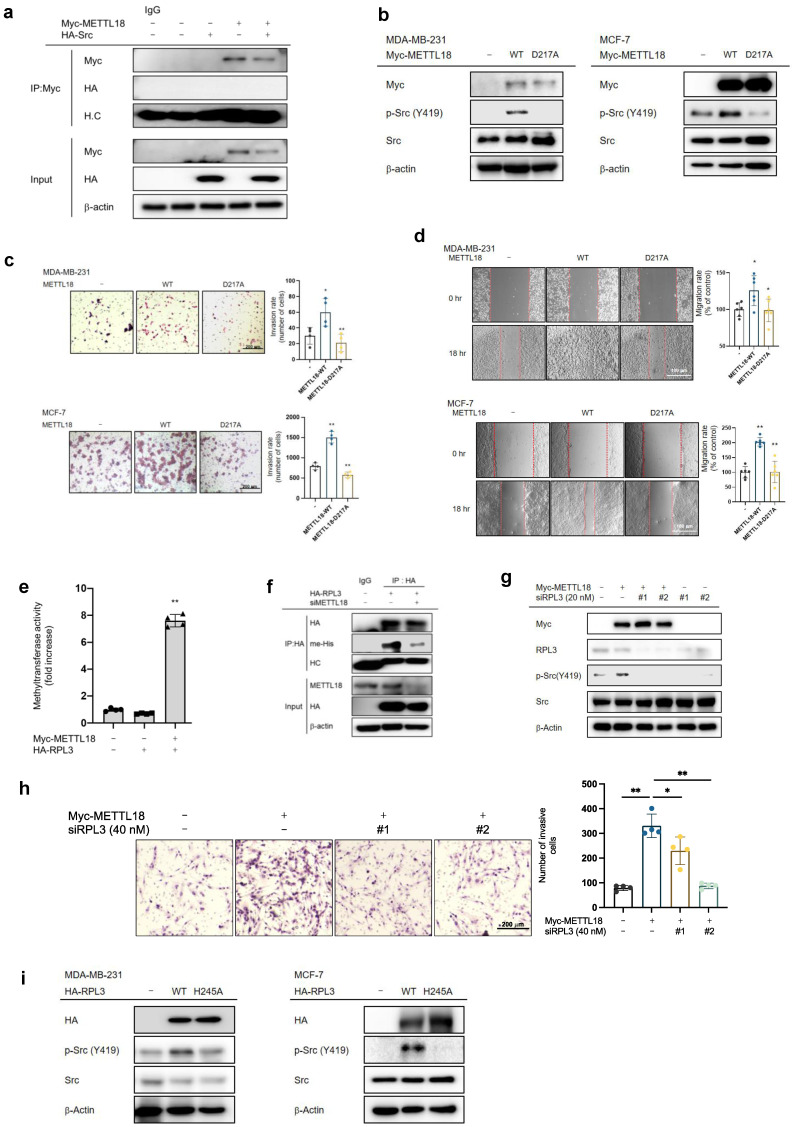
**Involvement of RPL3 in the METTL18-Src regulatory mechanism.** (A) Immunoprecipitation analysis of METTL18 and Src. The assay was performed with Myc, Myc-METTL18, HA, and HA-Src-overexpressing MDA-MB-231 cells. METTL18 was immunoprecipitated with anti-Myc followed by immunoblotting with anti-HA to identify Src. (B) Western blotting for the phospho- and total protein levels of Src in METTL18 WT- and METTL18 histidine 154 to lysine mutant (H154K)-transfected MDA-MB-231 cells and MCF-7. Transfection efficiency was tested by Western blotting with anti-Myc, and β-actin was used as the loading control. (C,D) The invasive capacity (C) and migrative capacity (D) of MDA-MB-231 cells and MCF-7 transfected with Myc, Myc-METTL18, or Myc-METTL18 H154K. The transfection efficacy of the plasmids and shRNA was verified by Western blotting with anti-Myc and anti-Src. (E) Methyltransferase assay with METTL18 and RPL3. For the *in vitro* methyltransferase assay, immunoprecipitated Myc and Myc-METTL18 prepared with lysates of MDA-MB-231 cells transfected with Myc-METTL18 or empty vector (Myc) were individually used as enzyme source. In addition, immunoprecipitated HA-RPL3 prepared with lysate of MDA-MB-231 cells transfected with HA-RPL3 or empty vector (HA) was used as a substrate. The enzyme and substrate sources were incubated with SAM (20 μM). Methyltransferase activity was determined via bioluminescence. (F) Histidine methylation level of RPL3 was evaluated with immunoprecipitated HA-RPL3 prepared with lysate of METTL18-knockdowned MDA-MB-231 cells transfected with HA-RPL3 or empty vector (HA) by immunoblotting with pan-anti-methylhistidine antibody. (G) Immunoblotting for the phospho- and total protein levels of Src in siRPL3- and METTL18-transfected MDA-MB-231 cells after 48 hours. (H) The invasion rate of MDA-MB-231 cells transfected with siRPL3 and METTL18. (I) Immunoblotting for the phospho- and total protein levels of Src in RPL3 WT and RPL3 histidine 245 to alanine mutant (H245A)-transfected MDA-MB-231 cells and MCF-7 cells. Transfection efficiency was tested by Western blotting with anti-HA, and β-actin was used as the loading control. The blots shown in (A), (B), (F), (G), and (I) are representative images of three independent Western blot experiments. ns: not significant; * *P* < 0.05; ** *P* < 0.01; ## *P* < 0.01.

We investigated the interaction of Src and METTL18 and observed no direct interaction (Fig. 4A). Moreover, our MALDI-TOF/MS analysis showed no histidine-methylated site in Src (data not shown). This suggests that Src is not a direct substrate of METTL18. In experiments using a METTL18 D217A catalytically inactive mutant, METTL18 WT induced the phosphorylation of Src, migration, and invasion, but the catalytically inactive METTL mutant did not show in both MDA-MB-231 and MCF-7 cells (Fig. 4B-D). These results imply that Src regulation depends on METTL18 methyltransferase activity, so we assessed whether RPL3, a known substrate of METTL18, can regulate the phosphorylation of the Src kinase. Firstly, we found that methylation of RPL3 can be increased by METTL18 according to our *in vitro* methylation assay (Fig. 4E). Overexpressed METTL18 increased the methylation level of HA-RPL3. In contrast, knockdown condition of METTL18 strongly reduced the histidine methylation level of HA-RPL3 (Fig. 4F). Secondly, we also tried to check whether RPL3 can affect the phosphorylation level of Src. Interestingly, siRPL3 suppressed the phosphorylation of Src induced by METTL18 overexpression in MDA-MB-231 cells (Fig. 4G). However, in the siRPL3 #2-transfected group (lane 6), a slight increase in p-Src Y419 levels was observed compared to the METTL18+siRPL3#2 group (lane 4). This discrepancy is likely attributable to the lower transfection efficiency of siRPL3#2. Moreover, the invasive capacity of breast cancer cells was reduced by siRPL3 in METTL18-overexpressing MDA-MB-231 cells (Fig. 4H). In addition, Src phosphorylation was induced by the overexpression of RPL3-WT but not by that of RPL3 H245A, a non-methylated mutant in both MDA-MB-231 and MCF-7 cells (Fig. 4I).

### Involvement of HSP90 and actin in the METTL18-RPL3-Src regulatory mechanism

**Figure 5:**
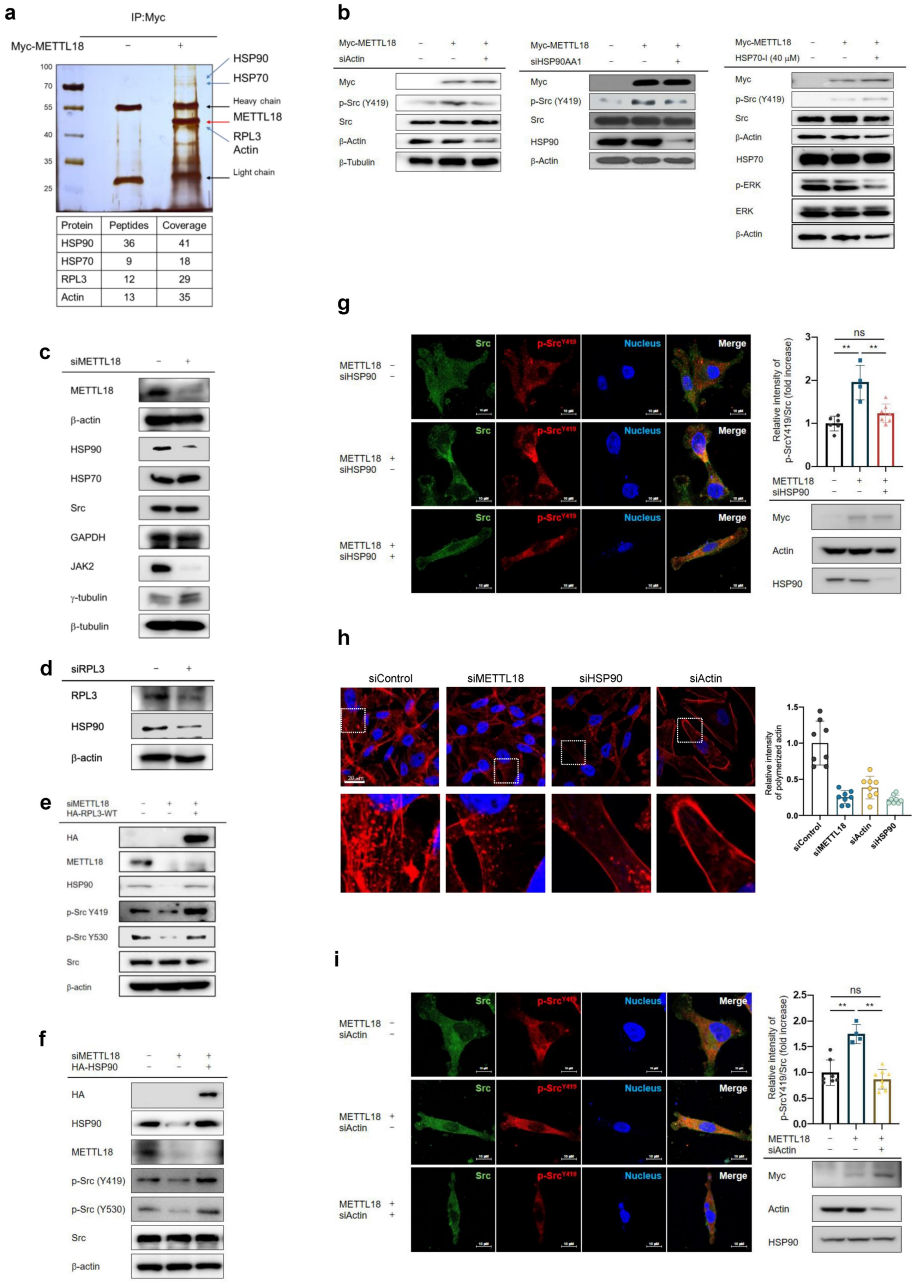
**Involvement of HSP90 and actin in the METTL18-Src regulatory mechanism.** (A) Silver staining image showing proteins immunoprecipitated with anti-Myc. The immunoprecipitation analysis was performed with whole lysates from Myc- or Myc-METTL18-overexpressing MDA-MB-231 cells, and each protein was identified by a further MS analysis. (B) Immunoblotting for p-Src and Src in MDA-MB-231 cells. Myc- or Myc-METTL18-overexpressing MDA-MB-231 cells were treated with siScramble, siHSP90AA1, and HEP70-I (VER15508). β-actin or β-tubulin was used as the loading control. The efficacy of the siRNAs was verified by immunoblotting with an antibody against each target protein. (C,D) Immunoblotting for HSP90 or JAK2 levels in the siScramble-, siMETTL18-, or siRPL3-transfected MDA-MB-231 cells. The transfection efficacy of the siRNA was examined by immunoblotting with anti-METTL18 and anti-RPL3. (E) Immunoblotting of HSP90, phospho-Src (Y419), phospho-Src (Y530), and Src in siScramble- or siMETTL18-transfected (48 hours) and HA-RPL3-WT-transfected (24 hours) MDA-MB-231 cells. The transfection efficacy of the siRNA and RPL3 was verified by Western blotting with anti-METTL18 and anti-HA. β-actin was used as the loading control. (F) Immunoblotting of HSP90, phospho-Src (Y419), phospho-Src (Y530), and Src in siScramble- or siMETTL18-transfected (48 hours) and HSP90-transfected (24 hours) MDA-MB-231 cells. The transfection efficacy of the siRNA and HSP90 was verified by Western blotting with anti-METTL18 and anti-HA. β-actin was used as the loading control. (G,I) Confocal microscopy images of Src (green) and phospho-Src (p-Y419) (red) in MDA-MB-231 cells transfected with Myc, Myc-METTL18, siScramble, siHSP90 (G), or siActin (I). Nuclei were stained with DAPI (blue). The transfection efficacy of each plasmid and siRNA was verified by immunoblotting with anti-Myc, anti-HSP90, and anti-actin. (H) Confocal microscopy images of polymerized actin (red) in MDA-MB-231 cells transfected with siScramble, siMETTL18, siHSP90, or siActin for 48 hours. Knockdown of METTL18, Actin, and HSP90 was confirmed by western blotting (Fig. S11A). Carl Zeiss Zen blue edition calculated the relative intensity of polymerized actin. The blots shown in (B), (C), (D), (E), (F), (G) and (I) are representative images of three independent Western blot experiments. ns: not significant; * *P* < 0.05; ** *P* < 0.01.

Next, we studied the binding partners of METTL18 to see whether other molecules are involved in the METTL18-RPL3-Src regulatory loop. We identified HSP90AA1, actin, and HSP70 as METTL18 interactors in an immunoprecipitation assay followed by an MS analysis (Fig. 5A). Of those proteins, actin and HSP90AA1 depletions reduced the Src phosphorylation induced by METTL18 to the control level (Fig. 5B). However, inhibiting HSP70 with VER15508 (HSP70-I) did not suppress p-Src levels (Fig. 5B). It has been reported that HSP70 activates ERK1/2, while VER-155008 decreases p-ERK levels 40,41. Based on these reports, we observed the p-ERK levels to demonstrate the inhibitory effect of VER-155008 on HSP70. As anticipated, VER-155008 significantly reduced p-ERK levels, indicating its proper functioning as an inhibitor of HSP70. The invasive capacity was also inhibited by siHSP90 and siActin in METTL18-overexpressing MDA-MB-231 cells (Fig. S8a and S8b). Moreover, in HER2-negative breast cancer patients, lower DMFS was observed in individuals with high expressions of HSP90 and β-actin (p=0.0006 and p=0.00035, respectively) (Fig. S9). In addition, we also found that tumor tissues expressing shMETTL18 RNA but not scrambled RNA showed reduced size of tumors (Fig. S9b), lowered levels of F-actin and p-Src (Fig. S9c and S9d). Recently, it has been reported that methylation of RPL3 at the histidine 245 residue slows tyrosine codon elongation, contributing to the quality and proteostasis of Tyr-rich proteins (proteins with more than 30 Tyr residues) such as JAK2 42. Consistent with that report, METTL18 knockdown reduced the JAK2 protein level here (Fig. 5C). Interestingly, a significant decrease in the HSP90 protein level was observed in both METTL18- and RPL3-depleted cells, while overexpression of METTL18 increased the protein level of HSP90 (Fig. 5C, D and Fig. S10A). In comparisons of protein levels utilizing a dataset from Breast Invasive Carcinoma (TCGA PanCancer Atlas), we consistently found a correlation between the protein levels of METTL18 and HSP90 (Table 1). However, the protein levels of CAMKMT, reported as an interactor with HSP90 43, and other METTL series proteins did not exhibit a significant positive correlation with HSP90 (Table 1). Most notably, the protein levels of HSP90 were elevated by RPL3 WT and were reduced by RPL3 H245A, a non-methylated mutant (Fig. S10B).

Meanwhile, the protein levels of other abundant proteins, such as actin, HSP70, GAPDH, Src, β-tubulin, and γ-tubulin, as well as the mRNA levels of HSP90AA, actin, HSP70, Src, β-tubulin, and γ-tubulin, remained unchanged following siMETTL18 treatment or METTL18 overexpression (Fig. 5C and Fig. S10A, and C). Additionally, siMETTL18 markedly suppressed the levels of HSP90, whereas the overexpression of HA-RPL3-WT restored the decreased levels of HSP90 (Fig. 5E). These results suggest that RPL3 methylation by METTL18 is an upstream event determining the quality of the HSP90 protein. The overexpression of RPL3 and HSP90 recovered the phosphorylation of Src in METTL18-knockdown MDA-MB-231 cells (Fig. 5E and F). Furthermore, in a confocal microscopy image analysis, the relative intensity of phospho-Src was increased in the METTL18 overexpression group and decreased in the shHSP90 groups (Fig. 5G). However, JAK2 did not recover the phosphorylation of Src in METTL18-inhibited MDA-MB-231 creased in the METTL18 overexpression group and decreased in the siHSP90 groups cells (Fig. S10D). These results imply that the METTL18-RPL3-HSP90 loop is responsible for regulating Src. Actin translation was independent of RPL3 activity, but our results suggest that actin might be involved in METTL18-mediated Src regulation. Previous reports suggest that HSP90 acts as the fundamental molecule of cell motility and invasion by modulating actin dynamics, and that actin polymerization regulates Src activation 44,45. Therefore, we investigated whether METTL18 and HSP90 affect actin polymerization. In confocal microscopy and the F/G actin assay, the polymerized actin level was decreased by siMETTL18, siHSP90, and siActin (Fig. 5H and Fig. S11B). METTL18 knockout in HAP-1 cells consistently led to decreased actin polymerization (Fig. S11C). Interestingly, the overexpression of HSP90 recovered the defect in siMETTL18-induced actin polymerization, suggesting a METTL18-HSP90-actin correlation (Fig. S11D). Furthermore, siActin reduced the phosphorylation of Src (Fig. 5I). According to the immunoprecipitation assay, actin binds to Src, and their interaction interferes with siMETTL18 (Fig. S11E). These results suggest that actin dynamics play a critical role in Src activation in a METTL18- and HSP90-dependent way. The effects of METTL18 on HSP90, actin, and Src were consistently observed in another HER2-negative breast cancer cell line, MCF-7 cells. shMETTL18 reduced the levels of HSP90 and p-Src (Y419) in MCF-7 cells (Fig. S11F). Additionally, shMETTL18-expressing MCF-7 cells exhibited disrupted actin polymerization (Fig. S11G).

### Importance of biphosphorylated Src in the METTL18-dependent Src activation pathway

**Figure 6:**
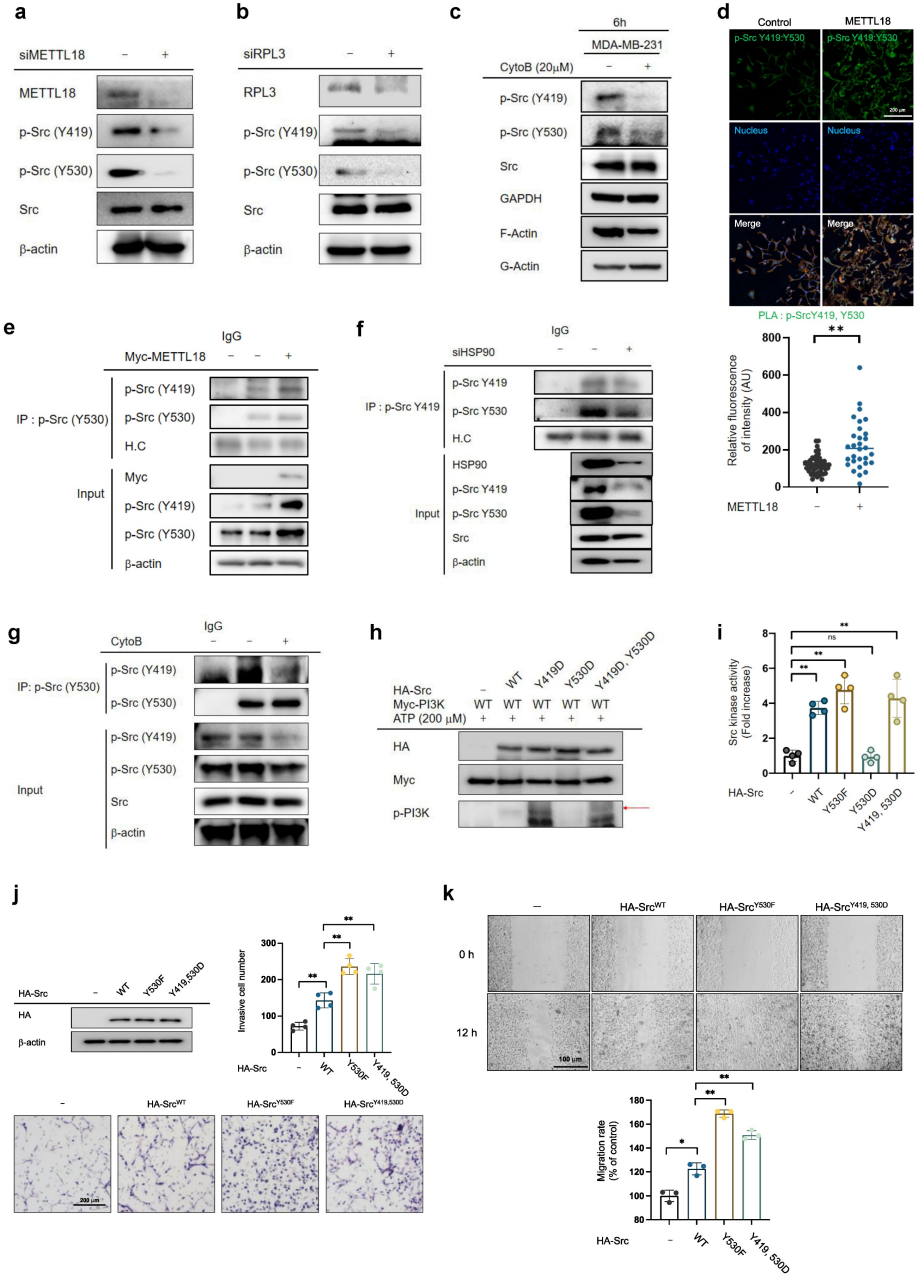
** Importance of biphosphorylated Src in METTL18-mediated Src activation.** (A,B) Immunoblotting for phospho-Src (Y419), phospho-Src (Y530), Src, and β-actin in siScramble-, siMETTL18- (A), and siRPL3- (B) transfected MDA-MB-231 cells. The transfection efficacy of the siRNA was tested by Western blotting with anti-METTL18 or anti-RPL3. (C) Immunoblotting of phospho-Src (Y419), phospho-Src (Y530), and Src in MDA-MB-231 cells treated with cytochalasin B (CytoB) for 6 hours. GAPDH serves as an internal reference. Additionally, the efficacy of CytoB was verified by observing the suppressed levels of filamentous actin (F-actin). (D) Confocal microscopy images showing DAPI and proximity ligation assay (PLA) signals in control and METTL18-expressing MDA-MB-231 cells. The cell nuclei were stained with DAPI (blue). The PLA was performed with primary antibodies against phospho-Src Y416 (rabbit polyclonal) and phospho-Src Y530 (mouse monoclonal), and the PLA signals are shown as green dots. The anti-p-Src Y416 was employed to detect human p-Src Y419. (E-G) Immunoprecipitation analysis to determine the presence of biphosphorylated Src kinase. We used lysates from MDA-MB-231 cells treated with Myc (E), Myc-METTL18 (E), siScramble (F), siHSP90 (F), or cytoB (G). Immunoprecipitation was performed with anti-phospho-Src (Y530), and each immunoprecipitation lysate was identified by immunoblotting with antibodies against phospho-Src (Y419) and phospho-Src (Y530). Heavy chain (H.C.) and β-actin were used as loading controls. Transfection and siRNA efficacy was identified by Western blotting with anti-Myc (e) or anti-HSP90 (f). (H,I) Kinase assay with Src WT and Src mutants. For the *in vitro* kinase assay (H), HA, HA-Src WT, HA-Src Y530F, HA-Src Y530D, and HA-Src Y419D/530D mutants were individually used as enzymes. In addition, Myc-PI3K was used as a substrate. The enzyme and substrate sources were prepared by immunoprecipitation and incubated with ATP (200 μM). Kinase activity was determined via immunoblotting against p-PI3K. Using a Src kinase enzyme system purchased from Promega (I), the assay was performed in HEK293 cells with immunoprecipitated HA, HA-Src WT, HA-Src Y530F, HA-Src Y530D, or HA-Src Y419D/530D. (J,K) The invasive capacity (J) and migrative capacity (K) of MDA-MB-231 cells transfected with HA, HA-Src WT, HA-Src Y530F, or HA-Src Y419/530D. Western blotting with anti-HA verified the transfection efficacy of the plasmids and shRNA. The blot shown in (A), (B), (C), (E), (F), (G), (H), and (J) is a representative image of three independent Western blot experiments. ns: not significant; * *P* < 0.05; # *P* < 0.05; ** *P* < 0.01; ## *P* < 0.01.

We questioned how the METTL18/RPL3/HSP90/actin axis regulates Src kinase. In canonical Src regulation, Src is present as p-Src(Y419), the active form, or p-Src(Y530), the inactive form. Interestingly, siMETTL18, METTL18 knockout, siRPL3, and cytochalasin B (an actin polymerization inhibitor) decreased both p-Y419 and p-Y530 levels in MDA-MB-231 cells and HAP-1 haploid cells (Fig. 6A, B, C, and Fig. S12), suggesting the existence of a novel form of the Src kinase. Indeed, we confirmed the presence of phosphorylated Src kinase at both sites (p-Src (Y419, Y530)) in two ways: 1) PLA for p-Src (Y419) and p-Src (Y530) (Fig. 6D) and 2) immunoprecipitation with p-Src (Y530) and immunoblotting with p-Src (Y419) (Fig. 6E). The overexpression of METTL18 induced phosphorylation of both p-Src Y419 and Y530 (Fig. 6D, E). Meanwhile, siHSP90AA1 and CytoB reduced p-Src (Y419, Y530) levels (Fig. 6F and G). These results imply that the generation of biphosphorylated Src is a critical event in the METTL18-dependent alternative Src regulatory pathway and a distinguishing feature of the canonical pathway.

The activity of the biphosphorylated Src kinase was examined using phosphomimetic mutants. The overexpression of Src-Y530F (a constitutively active form of Src) and Src-Y419D/Y530D (a biphosphorylated form of Src) showed higher kinase activity against PI3K than did Src-WT and high Src kinase activity in the Src kinase assay, whereas Src-Y530D (an inactive form of Src) did not (Fig. 6H and I). Furthermore, invasive and migrative capacities were elevated in Src-WT, Src-Y530F, and Src-Y419D/Y530D-overexpressing MDA-MB-231 cells (Fig. 6J and K). These results suggest that METTL18 generates Src biphosphorylated at Y419 and Y530 via kinase activity, increasing tumor metastasis.

## Discussion

Abnormal expression and activity of non-histone protein methyltransferases in tumors are recognized as crucial pathogenic factors. For example, low expression of EEF1AKMT3, which has a tumor suppressive function, correlates with poor prognosis of gastric cancer 46. In this study, we explored the biological function of METTL18 in cancer metastasis and dissected the molecular mechanisms underlying METTL18-mediated tumorigenic responses. Although other cancers, such as liver, lung, and bladder 47, are associated with high levels of METTL18, this study assessed the effect of METTL18 in breast cancer. The rationale for this focus on breast cancer stems from its clinical significance, particularly among patients with HER2-negative breast cancer (Fig. 1). Our results suggest that METTL18 plays a pivotal role in the metastatic process of HER2-negative breast cancer, and that the Src kinase is one of the intermediate molecules in the METTL18-mediated metastasis regulatory pathway. Src kinase is an oncoprotein, and the activation of Src induces the metastasis of breast cancer to the brain 48,49. Indeed, Src blockade shows promise for suppressing the metastatic potential of breast tumor cells. Furthermore, it has been reported that cell proliferation and migration in breast cancer were reduced under transfection of a construct with Src kinase-dominant negative form 50. Therefore, Src regulation via METTL18 suppression is expected to be a good strategy for HER2-negative breast cancer therapy.

Src lacks a methylated site of histidine (data not shown) and does not bind to METTL18 (Fig. 4A), implying that it is not a direct METTL18 substrate. Instead, we found the involvement of RPL3, a METTL18 substrate, in METTL18-mediated Src regulation. RPL3 is a component of the ribosome 60S subunit 20. The abnormal growth of cancer cells requires overactive translation, and emerging evidence suggests the presence of onco-ribosomes that promote oncogenic translational programs in cancer 51. It has also been reported that abnormal ribosomal proteins are associated with a poor cancer prognosis. For example, RPL3 is highly downregulated in colon cancer and promotes the progression of colorectal cancer cells 52. However, the role of RPL3 in breast cancer and its action mechanism are poorly understood. In our study, the loss of RPL3 and defects in its methylation inhibited p-Src levels, reducing the metastatic capacity of breast cancer cells. These results provide reliable evidence to establish the pathogenic role of RPL3 in breast cancers.

Previous reports revealed METTL18 interactors such as HSP90, HSP70, RPL3, and GRWD1 43. Similarly, we identified binding partners of METTL18, including HSP90, HSP70, RPL3, and actin. Among those binding partners, RPL3, HSP90, and actin were identified as participants in the activation of Src mediated by METTL18. In work by Falnes and co-workers, defects in RPL3 methylation derived from the loss of METTL18 caused rapid translation elongations on the Tyr codons at the A-site 42. Rapid ribosome elongation inhibits proper protein folding, resulting in low-quality proteins. As a result, Tyr-rich proteins, including JAK2 and DYNC2H1, are reported to aggregate and suffer proteasomal degradation in cells with RPL3 methylation defects 42. Interestingly, HSP90 protein levels were significantly decreased upon the loss of METTL18 and RPL3. HSP90, which has 25 Tyr residues, does not ideally fit the definition of a Tyr-rich protein, but our data support that HSP90 protein integrity might be determined by RPL3 methylation. This discrepancy with a previous report is likely because the specificity of the RPL3 effect might be influenced by factors (e.g., Tyr position) other than the number of Tyr residues. Collectively, our results suggest a METTL18-RPL3-HSP90-Src regulatory axis in breast cancer cells. While RPL3 methylation by METTL18 predominantly takes place in the nucleus 20, HSP90 is anticipated to promote Src phosphorylation by engaging robust interactions with METTL18 and actin, within the cytoplasmic compartment. Therefore, considering these spatial differences, it is inferred that METTL18, RPL3, and HSP90 do not form a terpolymer.

Several studies have highlighted an association between HER2 and HSP90 in HER2-positive breast cancer 53. Therefore, it could be anticipated that METTL18 contributes to the progression of HER2-positive breast cancer; however, our clinical data indicate that METTL18 is not associated with the prognostic outcomes of HER2-positive breast cancer. Consistent with the clinical data, siMETTL18 does not affect metastasis in HER2-positive SKBR3 cells (data not shown). The enhanced HSP90 observed in HER2-positive breast cancer does not seem to be a decisive factor contributing to the oncogenic potential of METTL18. Rather, the metastasis regulatory capability of METTL18 is presumed to arise from variances in its expression levels across different subtypes. Therefore, further investigation into the correlation between expressions of METTL18 and HER2 is warranted to elucidate the selective metastasis-promoting efficacy of METTL18 observed in HER2-negative breast cancer. Interestingly, downregulation of METTL18 in the MDA-MB-231 cells did not reduce the protein level of HER2 (data not shown), implying that METTL18 cannot directly modulate HSP90 by elevating HER2 status and rather lowered HER2 level might settle down the environment of increased level of METTL18 to manage oncogenic status of HER2-negative breast tumor cells. This possibility will be further examined in the following project. In addition, we posit that HER2 may influence METTL18 expression, underscoring the need for additional mechanistic investigations to clarify this association.

Subsequent experiments showed that actin proteostasis is independent of METTL18 and RPL3. Still, our results indicate that actin is a crucial regulator of the METTL18-Src pathway. We noted that siMETTL18 disrupted actin polymerization. The methylation of histidine-73 residues on actin is a critical determinant of actin polymerization 54, and SETD3, the sole actin histidine methyltransferase, has been reported to affect actin polymerization 18. However, we did not obtain clear evidence that METTL18 methylates actin and concluded that the fluctuations in actin polymerization observed in the METTL18-depleted model are independent of actin methylation. Our results demonstrate that the METTL18-HSP90-actin loop, which does not include the actin methylation process, contributes to Src regulation.

**Figure 7:**
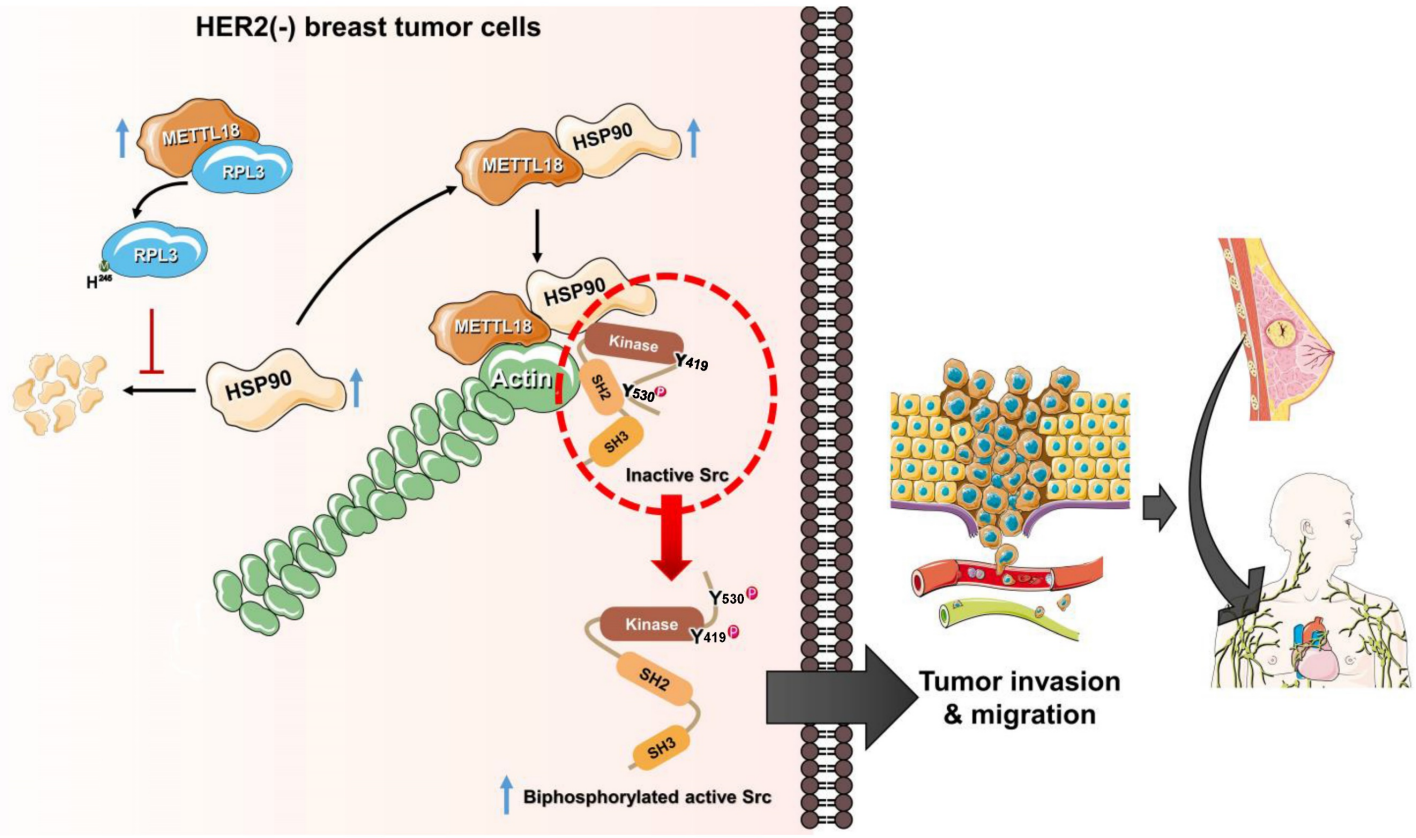
**The alternative Src regulatory pathway mediated by the METTL18-RPL3-HSP90-actin axis in HER2-negative breast cancers.** HER2-negative breast cancer expresses more METTL18 proteins than normal tissues and other breast cancer subtypes. METTL18 is responsible for RPL3 methylation at the histidine 245 residue, and that modification prompts the maintenance of HSP90 protein integrity. The increased HSP90 protein levels lead to actin polymerization. Actin filaments interact with the SH2 domain in the Src kinase, generating biphosphorylated Src with kinase activity. Ultimately, the biphosphorylated Src kinase increases the metastatic capacity of HER2-negative breast cancer, resulting in a poor prognosis.

Several reports indicate that actin is involved in Src regulation; indeed, in a previous study, we demonstrated that actin filaments modulate Src activity by binding to the kinase directly 29,45,55. However, the detailed molecular mechanism for actin-mediated Src activation remains to be fully elucidated. In this study, we propose an actin-Src regulatory mechanism as an alternative to the conventional mechanism. A key concept for the alternative regulation of Src by METTL18, HSP90, and actin is the generation of biphosphorylated Src. We suggest that the conformation of an autoinhibited Src kinase can be changed to an open state by binding to specific high-affinity molecules that can outperform intramolecular interactions to generate double p-Src 24. For example, FDGF 56 or FAK 57 binding to the SH2 domain of the Src kinase is associated with its activation. Similarly, actin can bind to the SH2 domain of Src 29, and the disruption of polymerized actin by siMETTL18 interfered with the interaction between actin and Src (Fig. S11D). The biphosphorylated Src had kinase activity similar to the conventional active form of the Src kinase; therefore, we argue that biphosphorylated Src is a crucial pathogenic molecule related to HER2-negative breast cancer with high METTL18 expression. Collectively, a novel Src regulatory model mediated by METTL18 is depicted in Fig. 7.

Interaction with chaperon proteins is a common feature among the 10 related human MTases: METTL21A, METTL21B, METTL21C, METTL21D, METTL23, METTL22, METTL20, EEF2KMT, CAMKMT, and METTL18. However, multiple pieces of evidence presented here strongly implicate a unique and specific interaction between METTL18 and HSP90 leading to the activation of Src kinase. First, although HSP70 is known to interact with METTL18, it was found not to be involved in the Src activation pathway. This is based on results observed with an HSP70 inhibitor (HSP70-I, Fig. 5B Left panel) showing no suppressive activity on Src phosphorylation. These results contrast with the HSP90 inhibitor, which showed inhibition of Src phosphorylation (Fig. S13A, B, and C), implying the specific role of HSP90 in METTL18-mediated Src regulation. Additionally, METTL18 showed clearly distinctive pattern compared with METTL21B, METTL22, and CAMKMT, which are also known as HSP90 interactants 43. METTL21B and METTL22 displayed tumor suppressive functions without activating Src (as assessed by colony formation, migration, and invasion assays) when Flag-METTL21A and Myc-METTL22 were overexpressed (Fig. S14A, B, C, and 15A, B). In contrast, CAMKMT and METTL18 both promoted tumor activity, but through different mechanisms: CAMKMT directly interacted with and likely methylated Src (detailed Src activation mechanisms by CAMKMT are currently being explored, data not shown), while METTL18 did not directly bind to Src (Fig. 4A). Our findings show that METTL18 forms a unique complex with HSP90, actin, and Src. Specifically, Src is not directly associated with METTL18 (Fig. 4A) unlike CAMKMT (Fig. S16E). Also, there was no direct binding between HSP90 and actin monomer (Fig. S17A). However, METTL18 was associated with both actin monomer (Fig. S17B) and HSP90 (Fig. S17C), while Src bound to both actin (Fig. S11D and S17D) via SH2 domain 29 and HSP90 (Fig. S17E). In particular, CTD domain of HSP90 was found as an important site in binding of METTL18 (Fig. S17F). Unlike CAMKMT, it is likely that METTL18 indirectly binds to Src but directly binds to translationally increased HSP90 (Fig. S10A). In addition, METTL18 and HSP90 were involved in regulation of polymerization of actin (Fig. 5H and Fig. S11A, B, and C), which is a critical event for Src phosphorylation in this molecular complex (Fig. 6G). Therefore, as we propose in Fig. 7, it is likely that there is a strong complex formation composed of METTL18, HSP90, actin, and Src, leading to specifically allowing the activation of Src via actin polymerization.

In conclusion, we demonstrated that the generation of biphosphorylated Src by a METTL18-driven RPL3/HSP90/actin/Src sequential pathway significantly contributes to the metastasis of HER2-negative breast cancer. HER2-negative breast cancers, including the luminal A and triple-negative types, account for 70-80% of all breast cancers 58. Patients with luminal breast cancer usually receive endocrine therapy (hormone therapy). However, resistance has been reported in 15-20% of cases due to either ERα-independent proliferative activation or phenotypic changes from ERα + to ERα - 59. In TNBC, systemic treatment efficiency is poor because of the absence of hormone receptors. In 2020, sacituzumab govitecan-hziy (brand name: Trodelvy) received FDA approval for TNBC patients 60, but other therapeutic regimens or targets are needed for patients who do not benefit from that drug. Therefore, we propose METTL18 as a valuable target molecule for the inhibition of metastasis and improvement of survival in HER2-negative breast cancer patients who receive no benefit from conventional drugs.

## Supplementary Material

Supplementary figures and tables.

## Figures and Tables

**Table 1:** Correlation of the protein expression level of METTL series and protein expression level of HSP90.

METTL series (Protein) vs. HSP90AA1 (Protein) (Pearson's correlation)					
Gene name	r	R2	P (two-tailed)	P value summary	Number of Pairs
METTL18	0.216	0.047	0.0271	*	105
TMT1B	0.199	0.039	0.1912	ns	45
METTL5	0.170	0.029	0.0827	ns	105
METTL6	0.132	0.017	0.3465	ns	53
METTL9	0.097	0.009	0.3568	ns	93
METTL17	0.058	0.003	0.5654	ns	102
METTL3	0.057	0.003	0.563	ns	105
METTL2B	0.010	0.000	0.9167	ns	105
CAMKMT	-0.1004	0.010	0.3462	ns	90
METTL14	-0.016	0.000	0.8685	ns	105
METTL25	-0.024	0.001	0.825	ns	84
METTL8	-0.053	0.003	0.6788	ns	64
METTL26	-0.205	0.042	0.0363	*	105
TRMT44	-0.292	0.085	0.0341	*	53
