# Supplementary material for: METTL18 functions as a Phenotypic Regulator in Src-Dependent Oncogenic Responses of HER2-Negative Breast Cancer
Source: Int J Biol Sci. 2024 Sep 3;20(12):4731–49. doi: 10.7150/ijbs.96487 (PMC11414398; doi:10.7150/ijbs.96487)

Supplementary information

METTL18 functions as a Phenotypic Regulator in Src-Dependent Oncogenic Responses of HER2-Negative Breast Cancer

Han Gyung Kim [1,+], Ji Hye Kim [1,+], Kyung-Hee Kim [2], Byong Chul Yoo [2], Sung-Ung Kang [3], Young Bong Kim [4], Sangmin Kim [5], Hyun-June Paik [6], Jeong Eon Lee [7], Seok Jin Nam [7], Narayanan Parameswaran [8], Jeung-Whan Han [9], Balachandran Manavalan [1,*], Jae Youl Cho [1,*]

[1]Department of Integrative Biotechnology, Sungkyunkwan University, Suwon 16419, Republic of Korea
[2]Research Institute, National Cancer Center, Goyang 10408, Republic of Korea.
[3]Neuroregeneration and Stem Cell Programs, Institute for Cell Engineering, Johns Hopkins University School of Medicine, Baltimore, MD 21205, USA
[4]Department of Bio-industrial Technologies, Konkuk University, Seoul 05029, Republic of Korea
[5]Breast Cancer Center, Samsung Medical Center, Seoul 06351, Republic of Korea
[6]Department of Surgery, Pusan National University Yangsan Hospital, Pusan National University School of Medicine, Yangsan 50612, Republic of Korea
[7]Division of Breast, Department of Surgery, Samsung Medical Center, Sungkyunkwan University School of Medicine, Seoul 06351, Republic of Korea
[8]Department of Physiology and Division of Pathology, Michigan State University, East Lansing, MI 48824, USA
[9]Research Center for Epigenome Regulation, School of Pharmacy, Sungkyunkwan University, Suwon 16419, Republic of Korea

Supplementary Figure legends

Figure S1. Clinical profile of METTL18 in breast tumor dataset. (A) The gene expression profiles of 23 methyltransferases, including METTL18, PKMTs, and PRMTs, in HER2-negative and HER2-positive breast cancer. We used the publicly available gene expression profiling interactive analysis 2 (GEPIA2) dataset (n=442). (B) Gene expression comparison of METTL13 and METTL18 in HER2-negative and HER2-positive breast cancer. TCGA data were used (n=1080). (C) Kaplan-Meier curve showing the survival probability of HER2-positive breast cancer patients with high or low expression of METTL18 (best cutoff). (D) DMFS of HER2-positive breast cancer patients with low or high expression of METTL18 (best cutoff).

Figure S2. (A) Immunoblotting of METTL18 protein in shScramble-, shMETTL18-, Myc, and METTL18 WT-transfected MDA-MB-231 cells. (B) Migration capacity of METTL18-knockdown MCF-7 cells.

Figure S3. Body weight of xenograft mice intravenously injected with shScramble- or shMETTL18-expressing MDA-MB-231 cells.

Figure S4. Signaling pathway activated by METTL18. (A) Immunoblotting for phospho- and total proteins, p65, p50, c-Jun, c-Fos, Syk, JAK2, and ATF25, in Myc-METTL18-overexpressing MDA-MB-231 cells. The transfection efficacy of the Myc-METTL18 construct was verified by immunoblotting with anti-Myc. β-actin was used as the loading control. (B) Immunoblotting of METTL18, p-Src (Y419), and Src expression in three types of breast cancer cells (SK-BR3, MDA-MB-453, and MDA-MB-231). β-actin was used as the loading control. ns: not significant; * $P < 0.05$; ** $P < 0.01$.

Figure S5. Levels of METTL18 and p-Src in breast tumor patients. Immunoblotting for phospho- and total Src and METTL18 in breast cancer patients from Samsung's cohort. β-actin was used as the loading control.

Figure S6. Gene expression levels of METTL18 and Src in breast tumor patients. Scatterplot showing the gene expression of Src and METTL18 in breast cancer patients from TCGA data. *P*-values and the correlation coefficient (*R*) were calculated using the Pearson test.

Figure S7. Role of Src in metastatic potential of breast tumor cells. (A,B) Invasion (A) and migration (B) ability of MDA-MB-231 cells transfected with shScramble or shSrc. (C) The transfection efficacy of the shRNA was verified by Western blotting with anti-Src. ** $P < 0.01$.

Figure S8. (A) Invasion ability of MDA-MB-231 cells transfected with siScramble, siHSP90AA1, or Myc-METTL18. (B) The invasive capacity of MDA-MB-231 cells transfected with siScramble, siActin, or Myc-METTL18. The transfection efficacy of siRNA and METTL18 was identified by immunoblotting with anti-HSP90AA1, anti-β-actin and anti-Myc. Invasive cell numbers were measured by ImageJ. ## $P < 0.01$; ** $P < 0.01$.

Figure S9. Relevance of involvement of HSP90, actin, and p-Src in human and mice. (A) Kaplan-Meier plots of distant relapse-free survival (DMFS) based on low or high expression of HSP90 and β-actin expressions in the HER2-negative breast cancer cohort (median cutoff) (GSE25066). (B) Tumor volume of MDA-MB2310 cells expressing scrambled RNA or shRNA to METTL18 (ShMETTL18). The long and short axes (D and d, respectively) of the tumors were determined using calipers. Tumor volume (mm^3) was subsequently estimated utilizing the formula: $V = 0.5 x D x d^2$. (C) F-actin level in sliced tumor tissues expressing shScramble RNA or shMETTL18 RNA from mice. (D). Level of p-Src from tumor tissues expressing shScramble or shMETTL18 RNA. * $P < 0.05$, ** $P < 0.01$, ns: not significant.

Figure S10. (A) Immunoblotting for Myc, β-actin, HSP90, HSP70, Src, GAPDH, JAK2, γ-tubulin, and β-tubulin in MDA-MB-231 cells transfected with METTL18 for 48 hours. (B) Immunoblotting for HSP90 in HA-RPL3 wild type or HA-RPL3 H245A overexpressing MDA-MB-231 cells. The transfection efficacy of the HA-RPL3 wild type and HA-RPL3-H245A construct was verified by immunoblotting with anti-HA. β-actin was used as the loading control. (C) The mRNA expression level of METTL18, HSP90AA, β-actin, HSP70, Src, β-tubulin and γ-tubulin was detected by quantitative real-time PCR in MDA-MB-231 cells transfected with siMETTL18 for 48 hours. GAPDH was used as a control gene. (D) Immunoblotting for JAK2, phospho-Src (Y419), phospho-Src (Y530), and Src in MDA-MB-231 cells transfected with siScramble, siMETTL18, or JAK2. siRNAs and plasmids were transfected for 48 hours and 24 hours, respectively. The transfection efficacy of the siRNA and JAK2 was verified by Western blotting with METTL18 and anti-JAK2. β-actin was used as the loading control.

Figure S11. (A) Immunoblotting of METTL18, HSP90, and Actin proteins in siScramble-, siMETTL18, siHSP90, and siActin-transfected MDA-MB-231. The observed decrease in HSP90 levels following siMETTL18 treatment is attributed to the diminished Rpl3 methylation. (B-D) Actin polymerization level of MDA-MB-231 and HAP-1 cells. (B,D) The F/G actin assay was performed with a G-actin/F-actin *in vivo* assay biochem kit (Cytoskeleton) in siHSP90 (B), siActin (B), siMETTL18 (D), or HSP90 (D)-transfected cells. (C) Confocal microscopy images showing polymerized actin (red) in HAP-1 wild type (WT) and HAP-1

METTL18 knockout (KO) cells. Carl Zeiss Zen blue edition calculated the relative intensity of the polymerized actin. (E) Immunoprecipitation analysis for interactions between actin and Src in siMETTL18-transfected MDA-MB-231 cells. (F-G) Effect of METTL18 in MCF-7 cells (F)Immunoblotting for p-Src (Y419), Src, and HSP90 in shMETTL18-expressing MCF-7. The transfection efficacy of the shMETTL18 was verified by Western blotting with anti-METTL18. β-actin was used as the loading control. (G) Confocal microscopy images of polymerized actin (Red) in shMETTL18-expressing MCF-7 cells. * $P < 0.05$, ** $P < 0.01$.

Figure S12. p-Src level of HAP-1 cells. Immunoblotting for HSP90, phospho-Src (Y419), phospho-Src (Y530), Src, and β-actin in HAP-1 wild type and HAP-1 METTL18 knockout cells. METTL18 knockout and transfection efficacy was tested by immunoblotting with anti-METTL18.

Figure S13. Pharmacological inhibition of HSP90AA1. (A) p-Src level of MDA-MB-231 cells transfected with Myc-METTL18 during 17-AAG treatment was analyzed by immunoblotting analysis. (B) Cell migration was examined with Myc-METTL18-overexpressed MDA-MB-231 cells treated with 17-AAG for 19 h. **c** Invasion level of MDA-MB-231 cells was evaluated under transfection of Myc-METTL18 and 17-AAG exposure.

Figure S14. Tumor-suppressive activity of METTL21B. (A) Colony formation was assessed by colony forming assay with Flag-METTL21B-overexpressed MKN-1 cells for 48 h. (B) Cell migration was examined with Flag-METTL21B-overexpressed MKN-1 cells treated for 24 h. **c** Invasion level of Flag-METTL21B-overexpressed MKN-1 cells for 24 h was evaluated by invasion assay. * $P < 0.05$, ** $P < 0.01$.

Figure S15. Tumor-suppressive activity of METTL22. (A) Cell migration was examined with Myc-METTL22-overexpressed HCT-116 cells treated for 24 h and 48 h. (B) Invasion level of Myc-METTL22-overexpressed HCT-116 cells for 48 h was evaluated by invasion assay. ** $P < 0.01$.

Figure S16. Tumor promoting activity of CAMKMT. (A) Colony formation was assessed by colony forming assay with Myc-CAMKMT-overexpressed MKN-1 cells for 48 h. (B,C) Invasion levels of Myc-CAMKMT-overexpressed (B) or shCAMKMT-expressing (C) MKN-1 cells for 24 h were evaluated by invasion assay. (D) Levels of p-Src was confirmed by immunoblotting analysis with lysates of Myc-CAMKMT-overexpressed or shCAMKMT-expressing MKN-1 cells. (D) Level of complex formation between CAMKMT and Src was detected by immunoblotting analysis with beads prepared by immunoprecipitation with anti-Myc with lysates of MKN-1 cells transfected with Myc-CAMKMT and/or HA-Src. * $P < 0.05$, ** $P < 0.01$.

Figure S17. Molecular complex formation between METTL18, HSP90, actin and Src. (A-F) Levels of complex formation between actin and HSP90 (A), METTL18 and actin (B), METTL18 and HSP90AA1 (C), actin and Src (D), HSP90 and Src (E), and METTL18 and HSP90 (F) were detected by immunoblotting analysis with beads prepared by immunoprecipitation with anti-GFP, anti-Flag, and anti-HA, with lysates of MDA-MB-231 cells transfected with GFP-Actin, HA-Actin, HA-HSP90, Flag-METTL18, or HA-Src. **g** Construct map of HSP90AA1 deletion mutation.

Supplementary Tables

Table S1. List of primers used for siRNA against METTL18, HSP90AA1, actin, PIMT, EEF2KMT, PRMT1, GRWD1, and RPL3.

Name of siRNA	Forward (5'-3')	Reverse (5'-3')
METTL18	CCAGAUUAUUAUAGUAAUUUU	AAUUACUAUAAUAAUCUGGUU
HSP90AA1	GAAACAUUCUCAGUUUAUUUU	AAUAAACUGAGAAUGUUUCUU
Actin	CGAGAAGAUGACCCAGAUCAUUU	AUGAUCUGGGUCAUCUUCUCGUU
PIMT	CUCGGAGCUAAUCCACAAUUU	AUUGUGGAUUAGCUCCGAGUU
EEF2KMT	CUUAGAAGCAAAGUUAAGAUU	UCUUAACUUUGCUUCUAAGUU
PRMT1	CGUCAAAGCCAACAAGUUAUU	UAACUUGUUGGCUUUGACGUU
GRWD1	GGGAUGAGCAGGCCCAAAUGAAGCCUU	GGCUUCAUUUGGGCCUGCUCAUCCCUU
RPL3	CCAAGUCAUCCGUGUCAUUUU	AAUGACACGGAUGACUUGGUU
Scramble	CCUACGCCACCAAUUUCGUUU	ACGAAAUUGGUGGCGUAGGUU

Table S2. List of primers used to produce shRNA constructs against the METTL18 and Src genes.

Name of shRNA	Forward (5'-3')	Reverse (5'-3')
shMETTL18	CCGGCATTTACAACCCAGATTATTACTCGAGTAATAATCTGGGTTGTAAATGTTTTTG	AATTCAAAAACATTTACAACCCAGATTATTACTCGAGTAATAATCTGGGTTGTAAATG
shSrc #1	CCGGGCTCGGCTCATTGAAGACAATCTCGAGATTGTCTTCAATGAGCCGAGCTTTTTG	AATTCAAAAAGCTCGGCTCATTGAAGACAATCTCGAGATTGTCTTCAATGAGCCGAGC
shSrc #2	CCGGGACAGACCTGTCCTTCAAGAACTCGAGTTCTTGAAGGACAGGTCTGTCTTTTTG	AATTCAAAAAGACAGACCTGTCCTTCAAGAACTCGAGTTCTTGAAGGACAGGTCTGTC

Fig. S1a

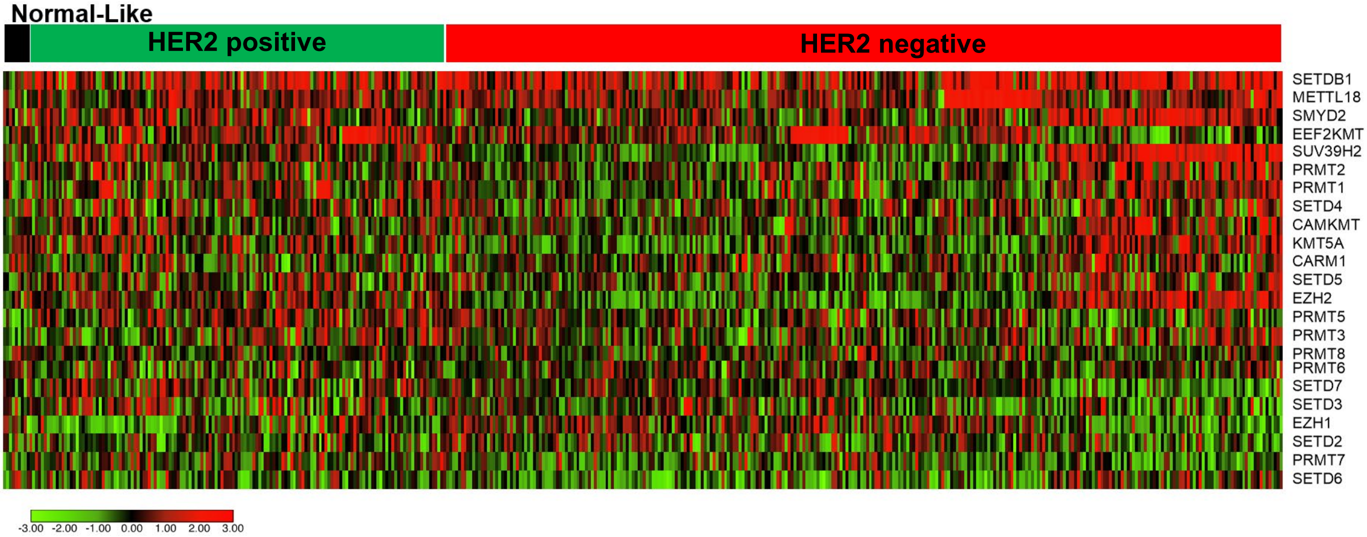

Fig. S1b

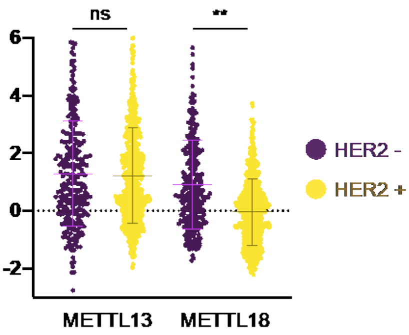

Fig. S1c

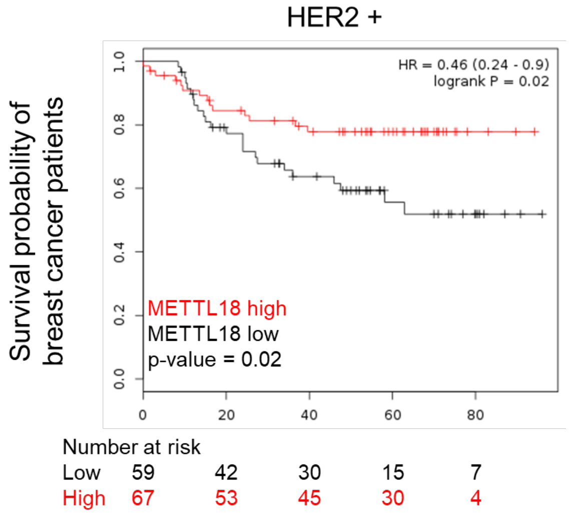

Fig. S1d

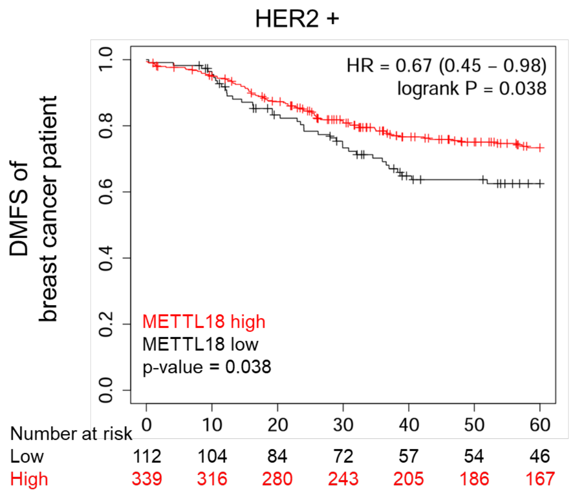

Fig. S2a

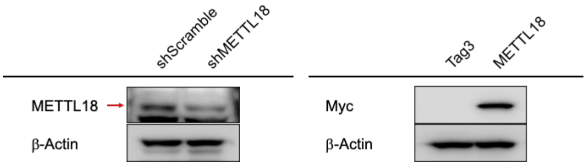

Fig. S2b

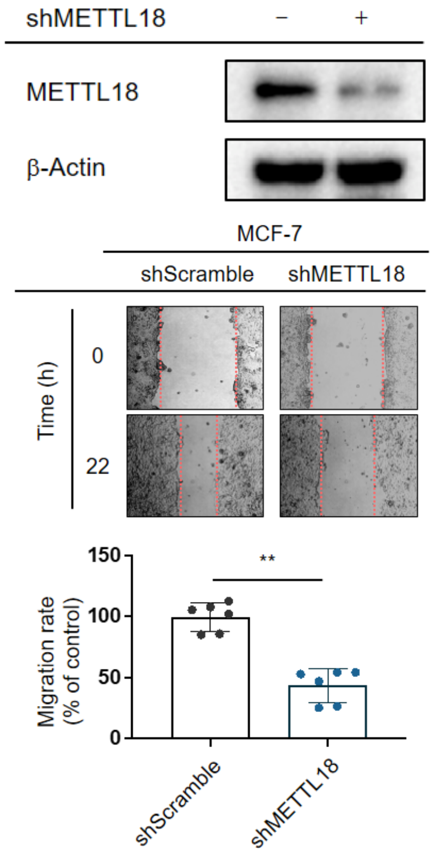

Fig. S3

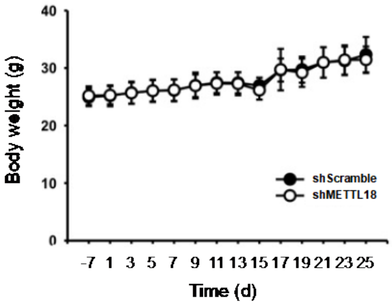

Fig. S4a

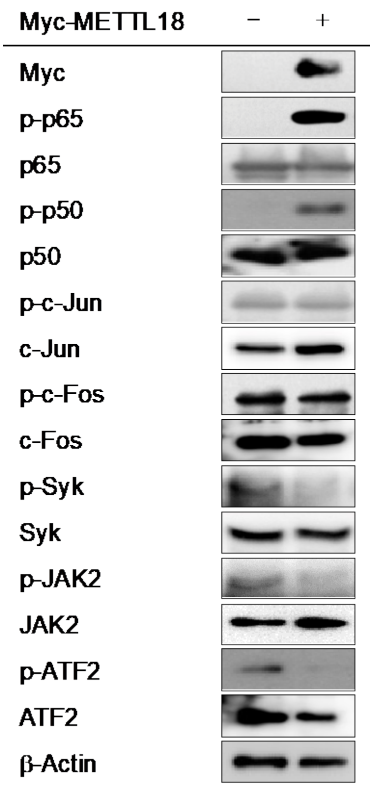

Fig. S4b

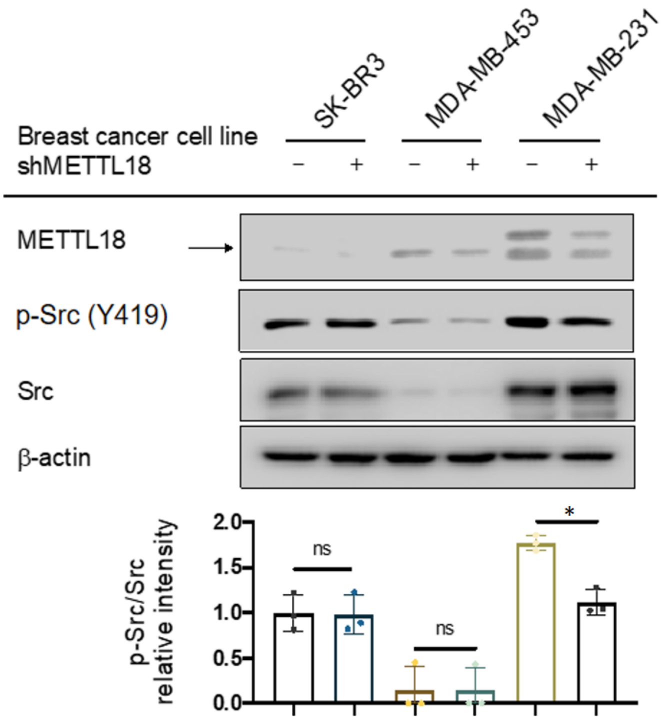

Fig. S5

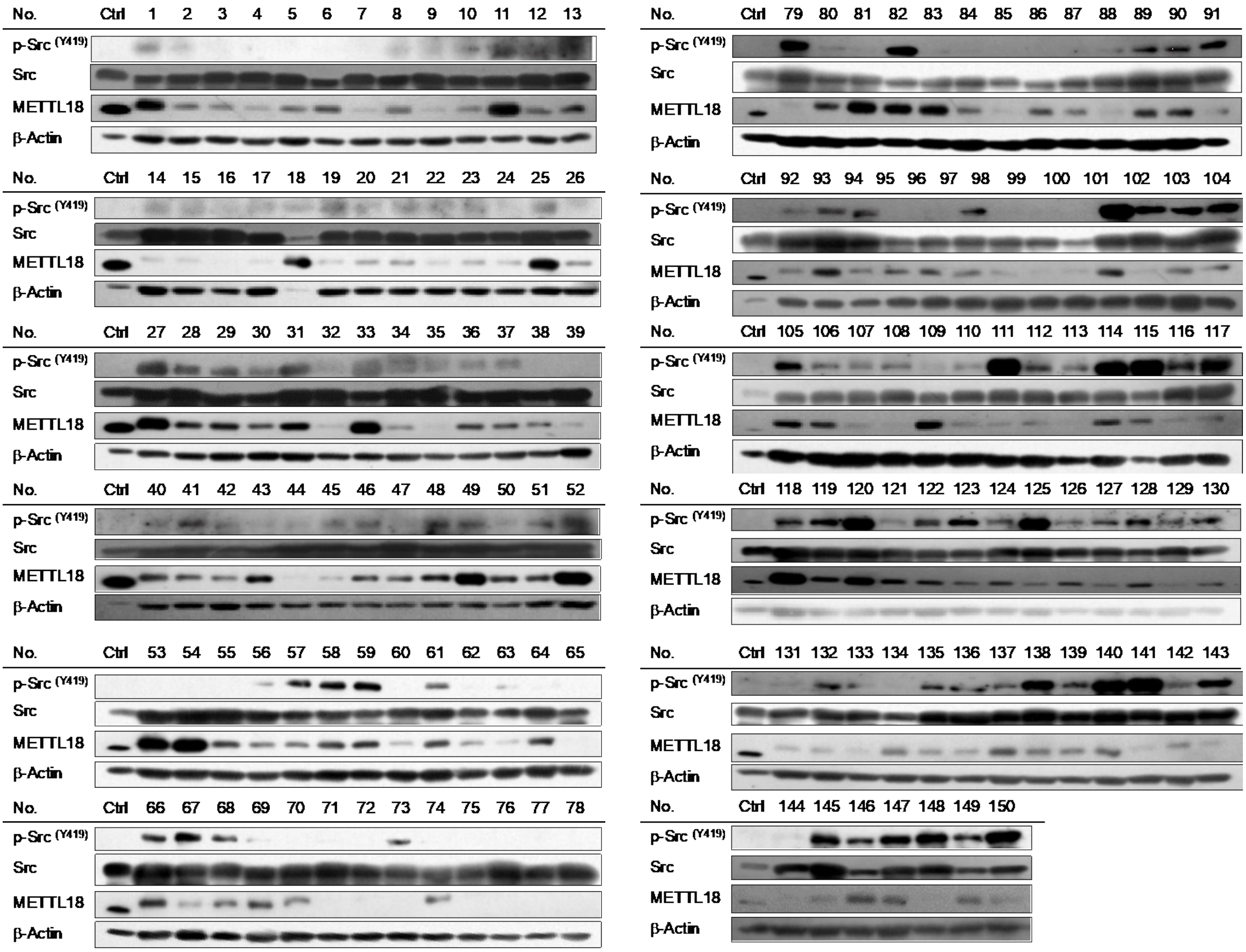
No. Ctrl 1 2 3 4 5 6 7 8 9 10 11 12 13
p-Src (Y419)
Src
METTL18
β-Actin
No. Ctrl 14 15 16 17 18 19 20 21 22 23 24 25 26
No. Ctrl 27 28 29 30 31 32 33 34 35 36 37 38 39
No. Ctrl 40 41 42 43 44 45 46 47 48 49 50 51 52
No. Ctrl 53 54 55 56 57 58 59 60 61 62 63 64 65
No. Ctrl 66 67 68 69 70 71 72 73 74 75 76 77 78
No. Ctrl 79 80 81 82 83 84 85 86 87 88 89 90 91
No. Ctrl 92 93 94 95 96 97 98 99 100 101 102 103 104
No. Ctrl 105 106 107 108 109 110 111 112 113 114 115 116 117
No. Ctrl 118 119 120 121 122 123 124 125 126 127 128 129 130
No. Ctrl 131 132 133 134 135 136 137 138 139 140 141 142 143
No. Ctrl 144 145 146 147 148 149 150

Fig. S6

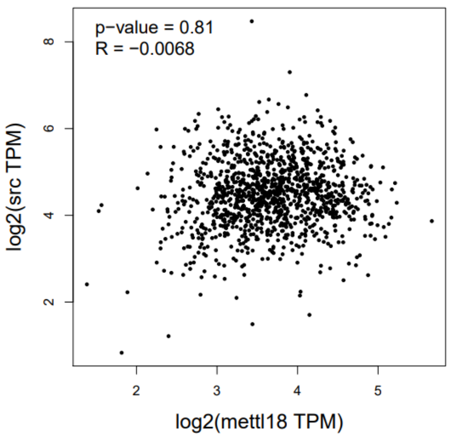

Fig. S7a

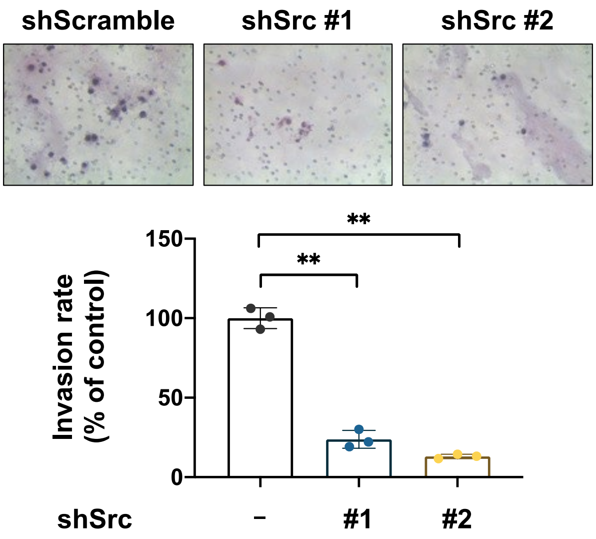

Fig. S7b

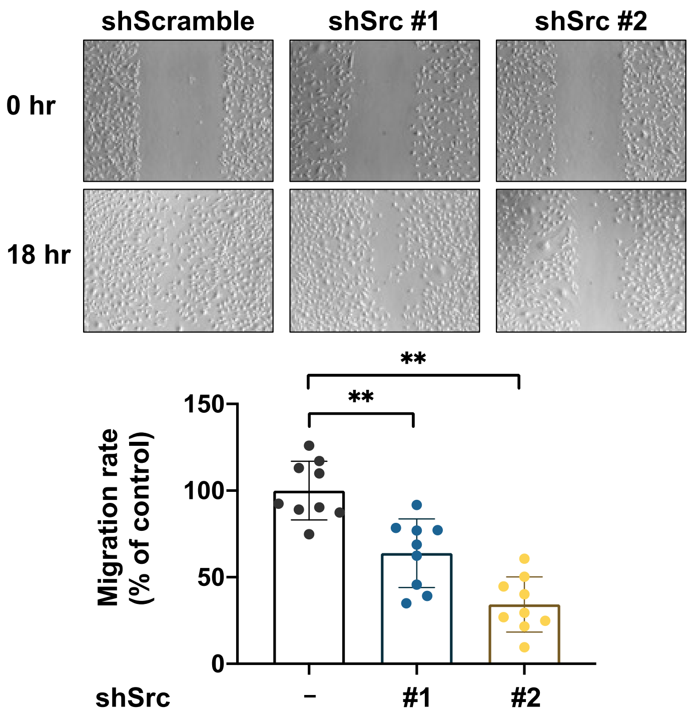

Fig. S7c

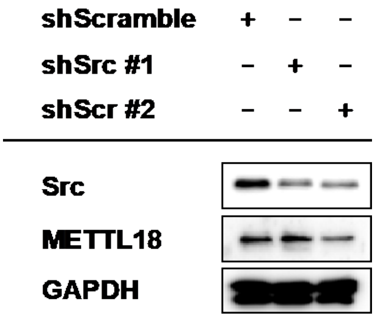

Fig. S8a

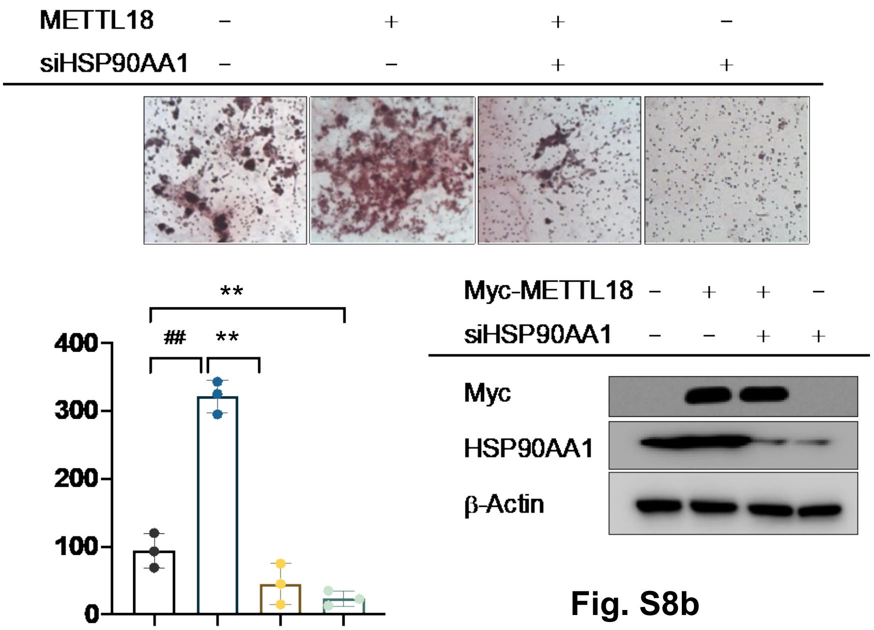

Fig. S8b

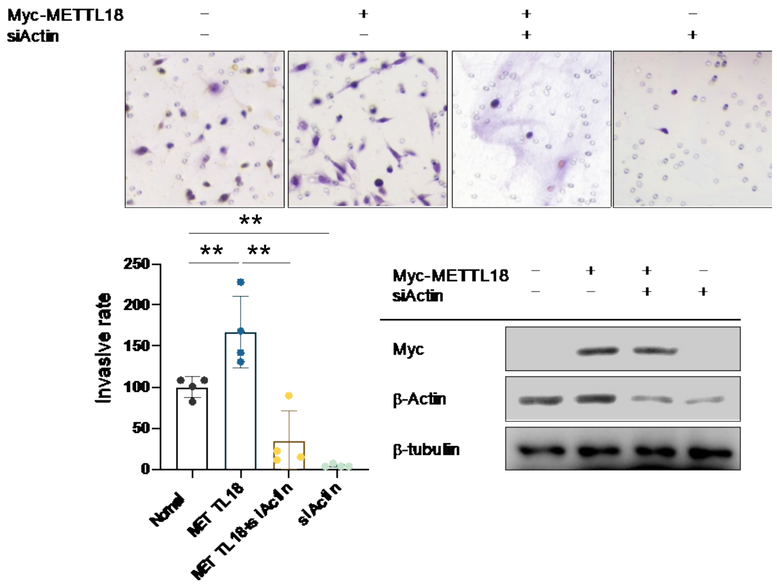

Fig. S9a

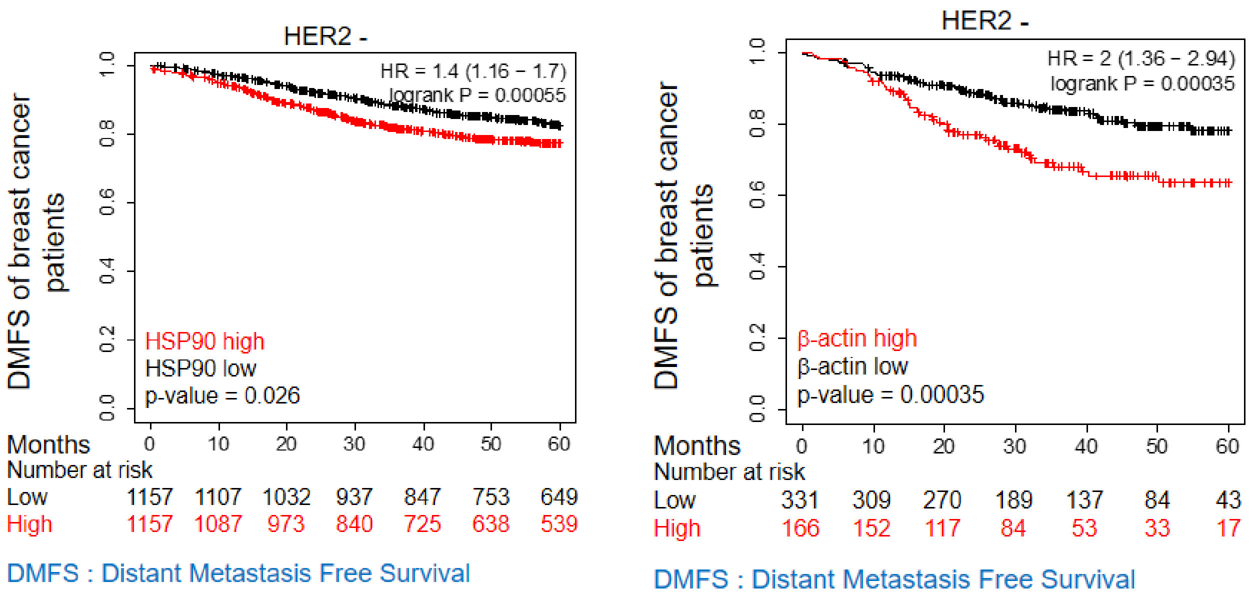

Fig. S9b

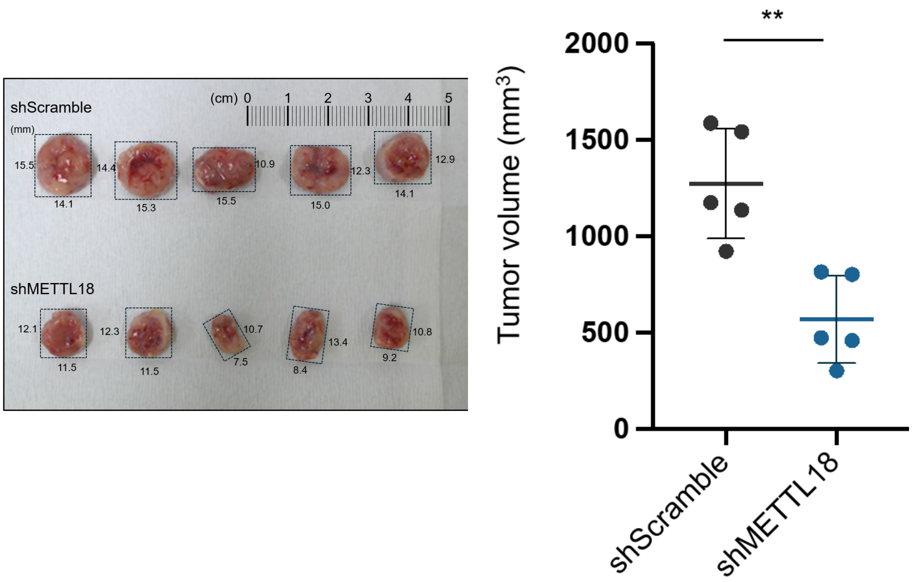

Fig. S9c

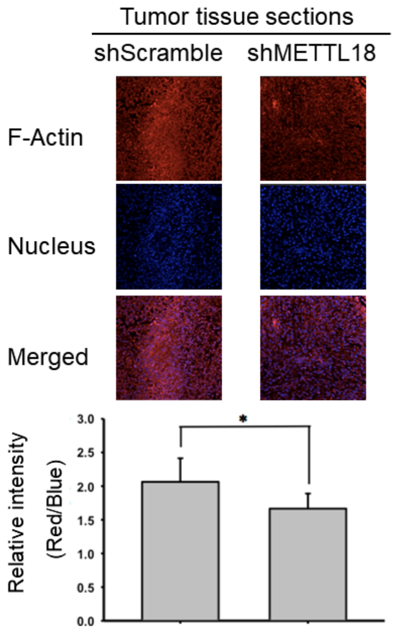

Fig. S9d

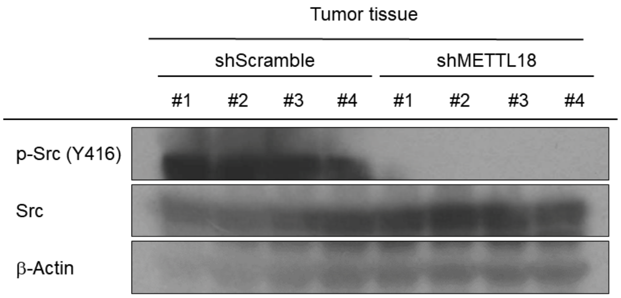

Fig. S10a

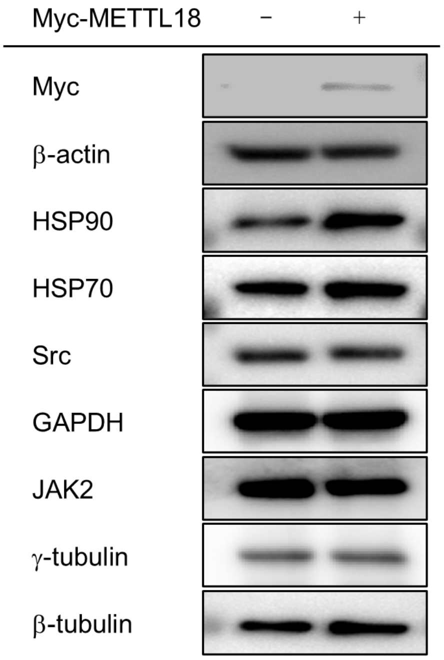

Fig. S10b

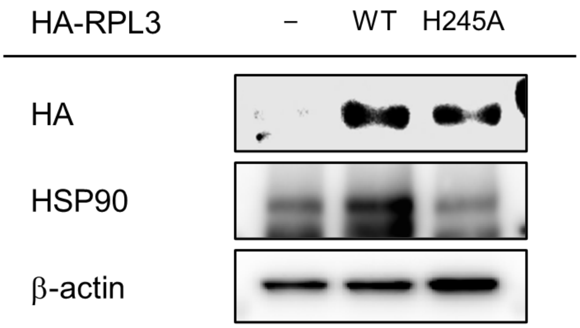

Fig. S10c

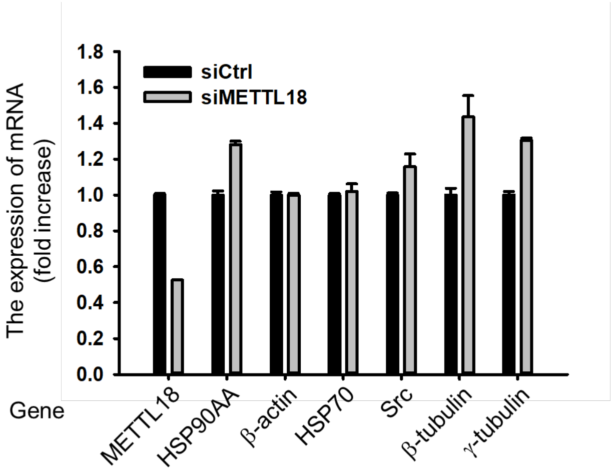

Fig. S10d

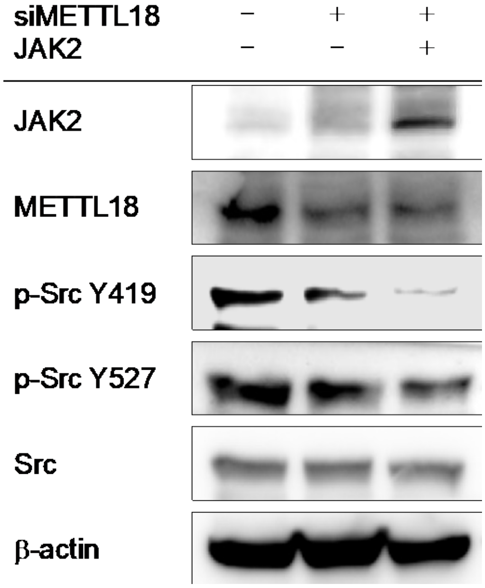

Fig. S11a

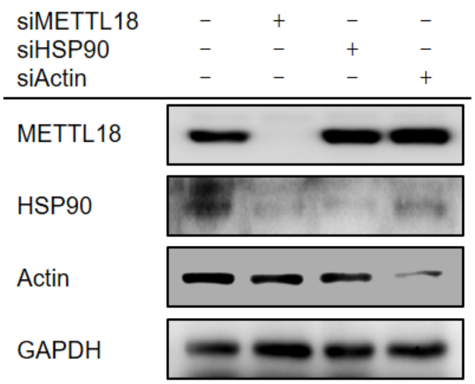

Fig. S11b

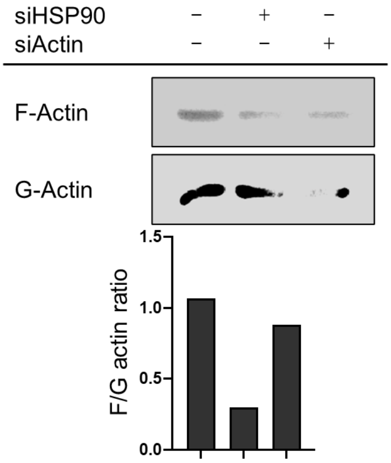

Fig. S11d

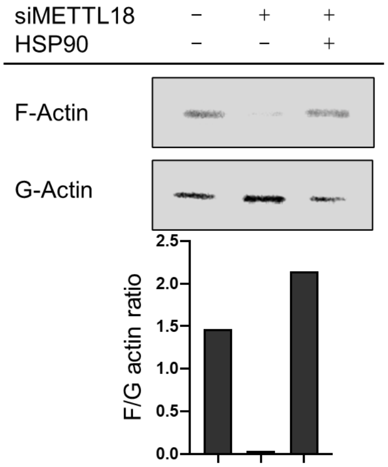

Fig. S11c

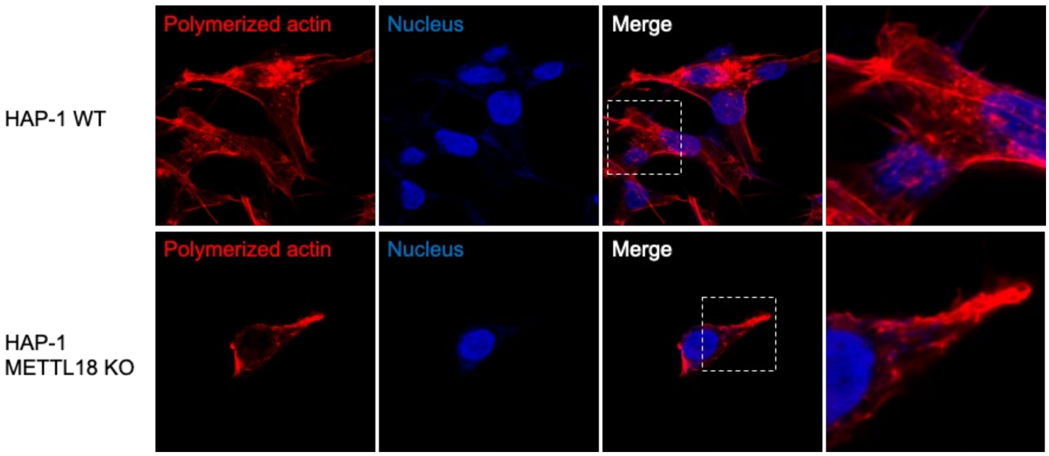

Fig. S11e

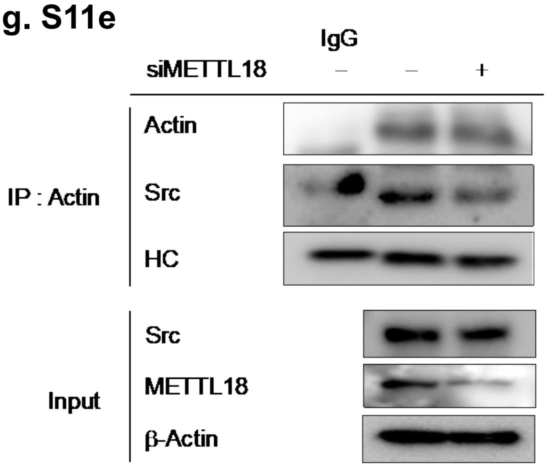

Fig. S11f

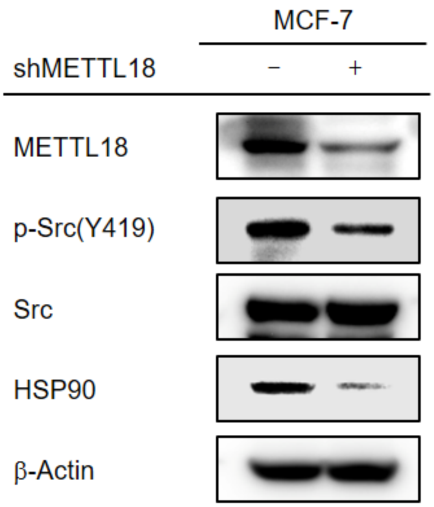

Fig. S11g

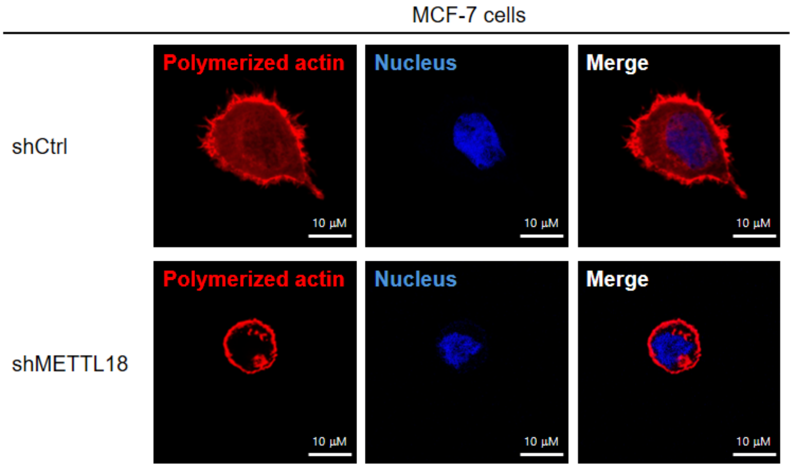

Fig. S12

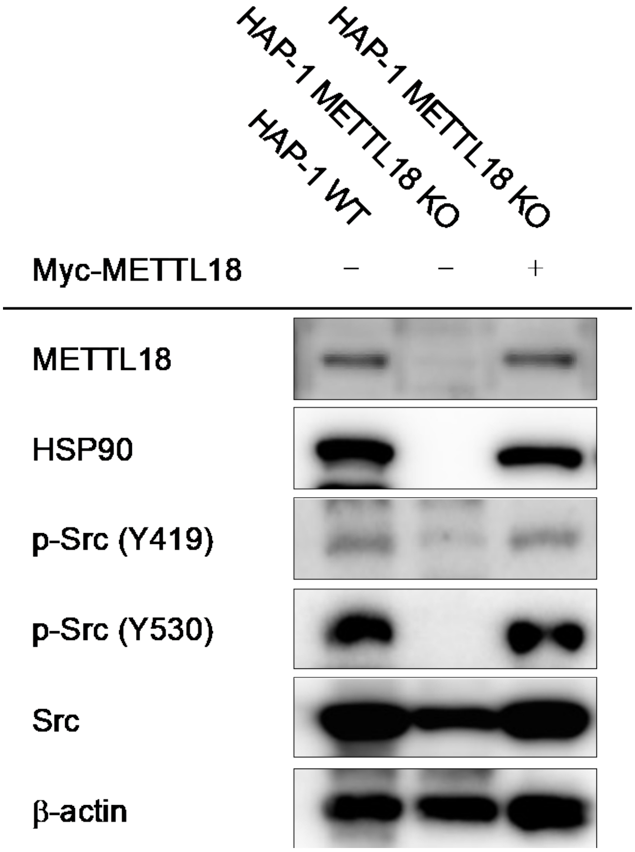

Fig. S13a

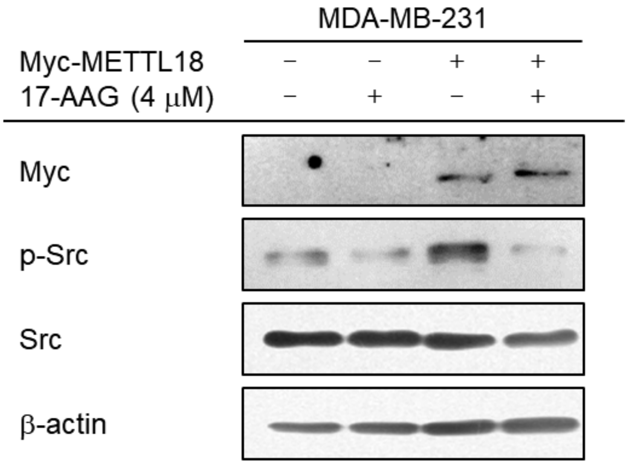

Fig. S13b

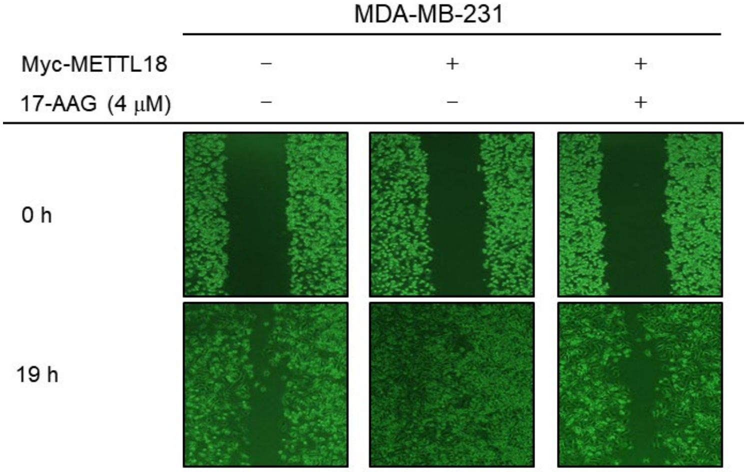

Fig. S13c

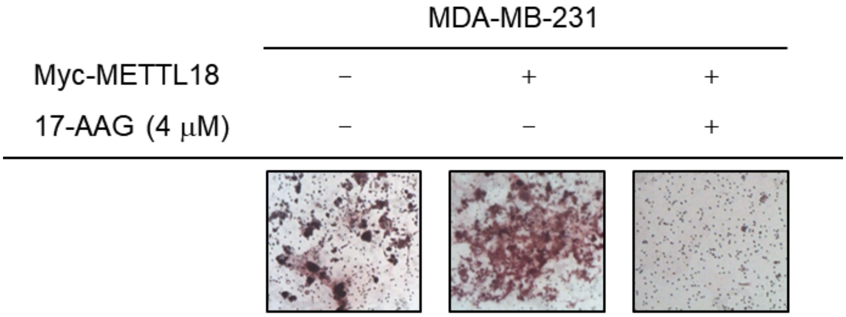

Fig. S14a

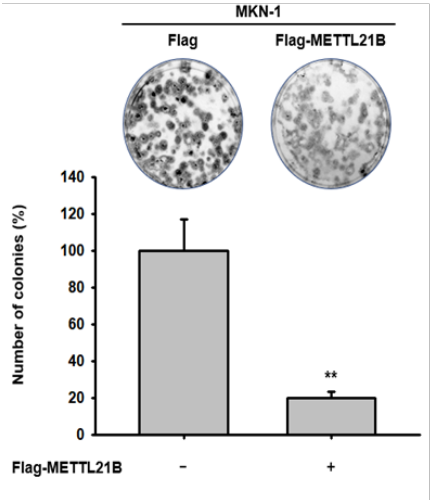

Fig. S14b

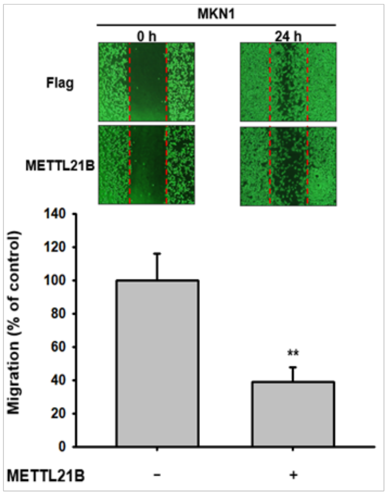

Fig. S14c

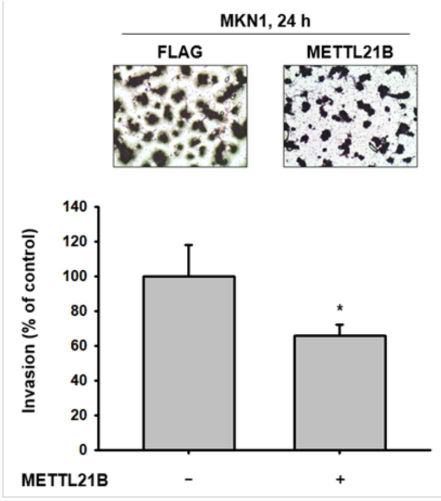

Fig. S15a

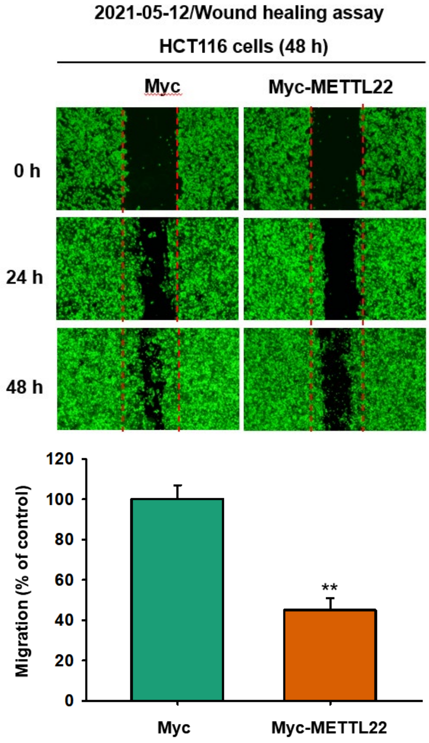

Fig. S15b

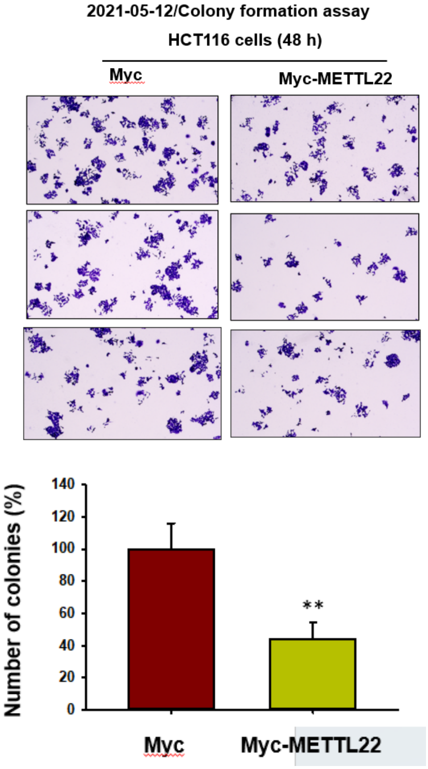

Fig. S16a

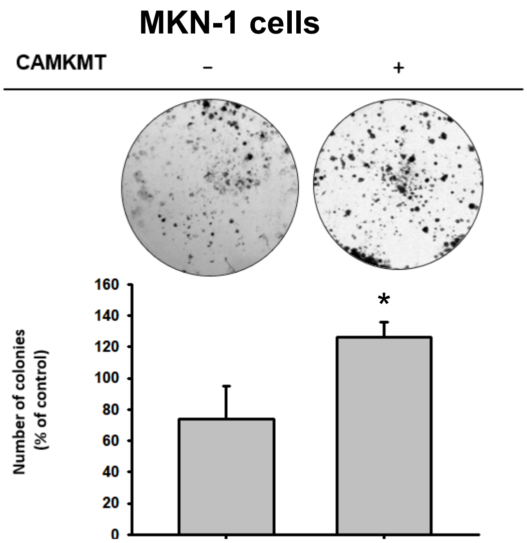

Fig. S16b

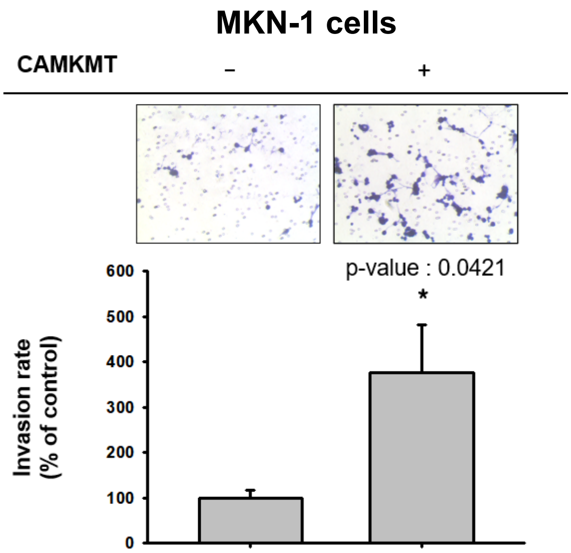

Fig. S16c

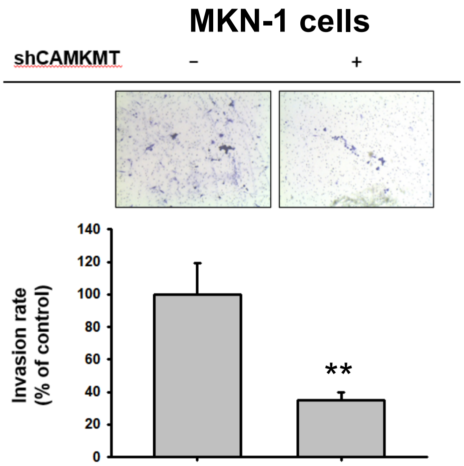

Fig. S16d

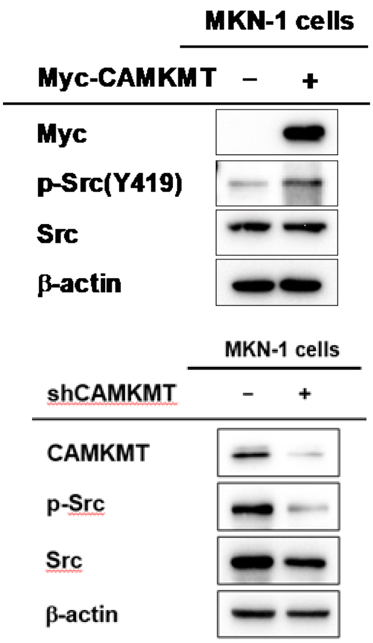

Fig. S16e

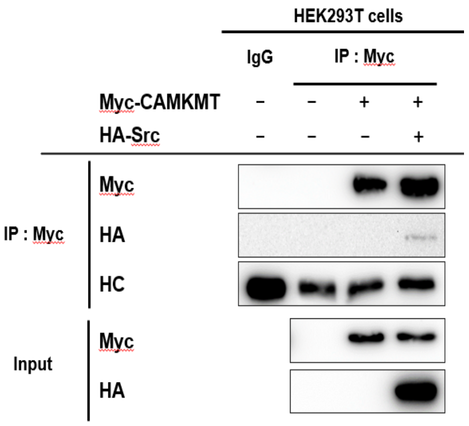

Fig. S17a

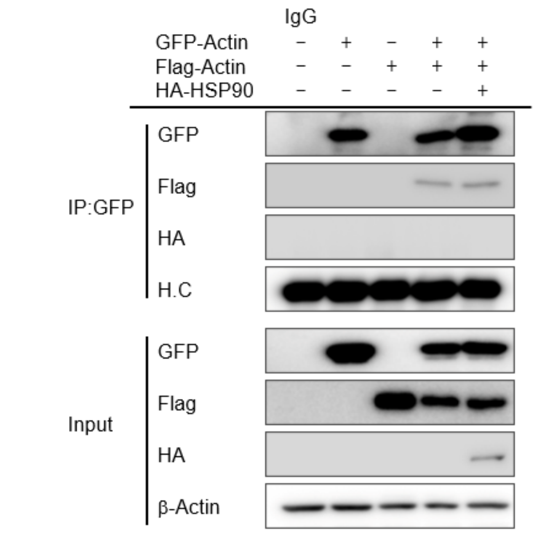

Fig. S17c

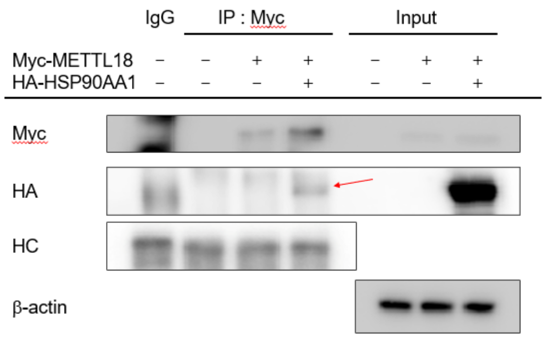

Fig. S17e

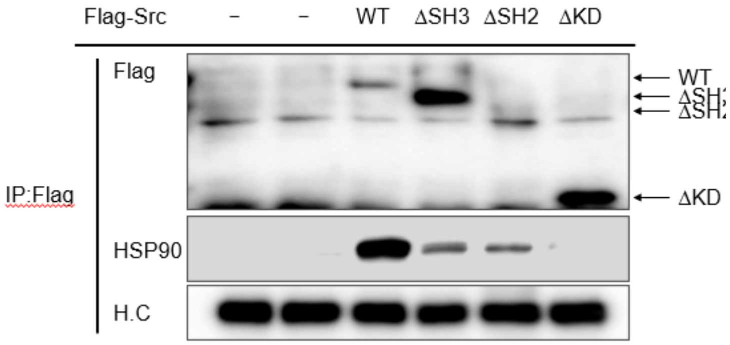

Fig. S17b

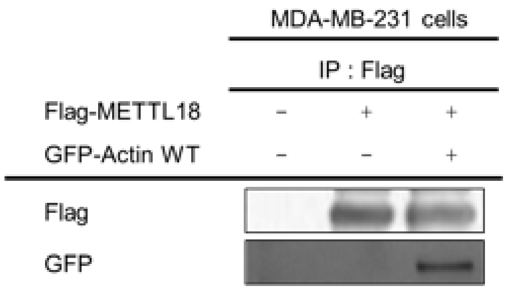

Fig. S17d

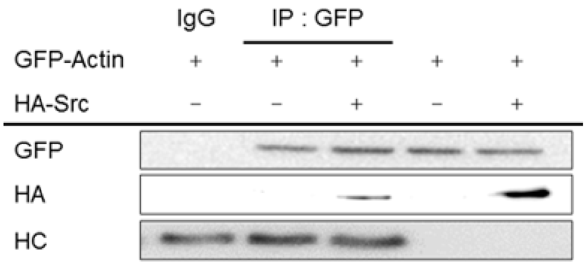

Fig. S17f

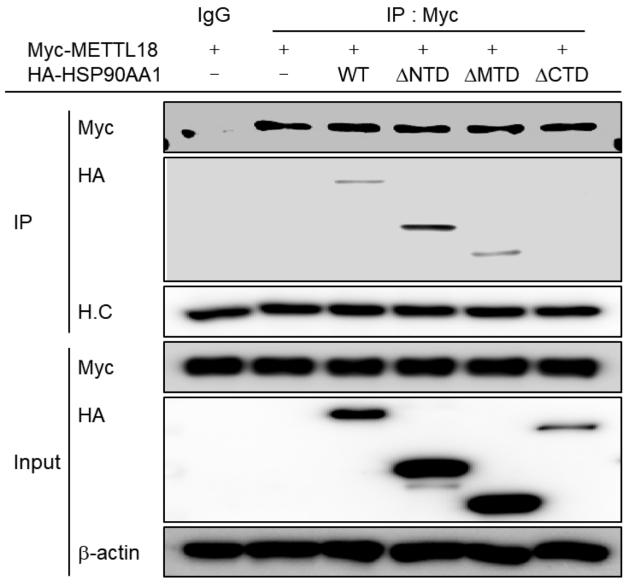

Fig. S17g

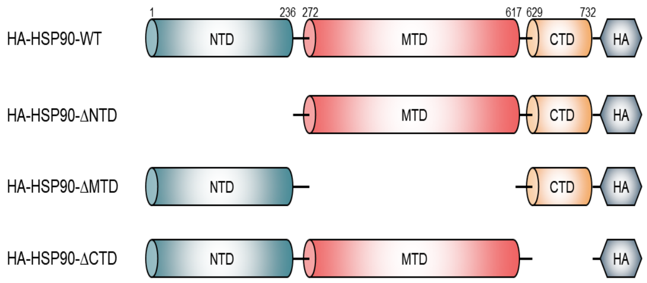

Supplement: Supplementary file 1 — Supplementary figures and tables. [file ijbsv20p4731s1.pdf]
